# Effect of catalase on CPC production during fermentation of *Acremonium chrysogenum*

**DOI:** 10.1186/s40643-024-00831-y

**Published:** 2025-01-04

**Authors:** Ling Liu, Zhen Chen, Xiwei Tian, Ju Chu

**Affiliations:** Qingdao Innovation Institute of East China University of Science and Technology, State Key Laboratory of Bioreactor Engineering, East China University of Science and Technology, 130 Meilong Road, Shanghai, 200237 People’s Republic of China

**Keywords:** Cephalosporin C, *A. chrysogenum*, Surfactants, Catalase, ROS

## Abstract

Cephalosporin C (CPC) is a critical raw material for cephalosporin antibiotics produced by *Acremonium chrysogenum*. During fermentation, the oxygen supply is a crucial factor limiting the efficient biosynthesis of CPC. This study demonstrated that the addition of exogenous surfactants significantly increased the dissolved oxygen (DO) level, extracellular catalase content, and final CPC titer. Consequently, we hypothesized and examined a correlation between catalase and CPC biosynthesis in *A. chrysogenum* through both the exogenous addition of hydrogen peroxide (H₂O₂) and the endogenous modulation of the *catA* expression level. The results indicated that both the addition of H₂O₂ and the ∆*catA* mutation exhibited similar fermentation trends, leading to decreased extracellular catalase activity and increased intracellular reactive oxygen species (ROS) content, which resulted in reduced CPC production. Conversely, strains that overexpress varying levels of the *catA* accelerated hyphal differentiation under DO-limiting conditions, reducing intracellular ROS accumulation and decreasing cellular apoptosis, which stabilized CPC yield during the later stages of fermentation. This study provides a critical foundation for further investigations into the regulatory mechanisms governing CPC biosynthesis in *A. chrysogenum*.

**Graphical Abstract:**

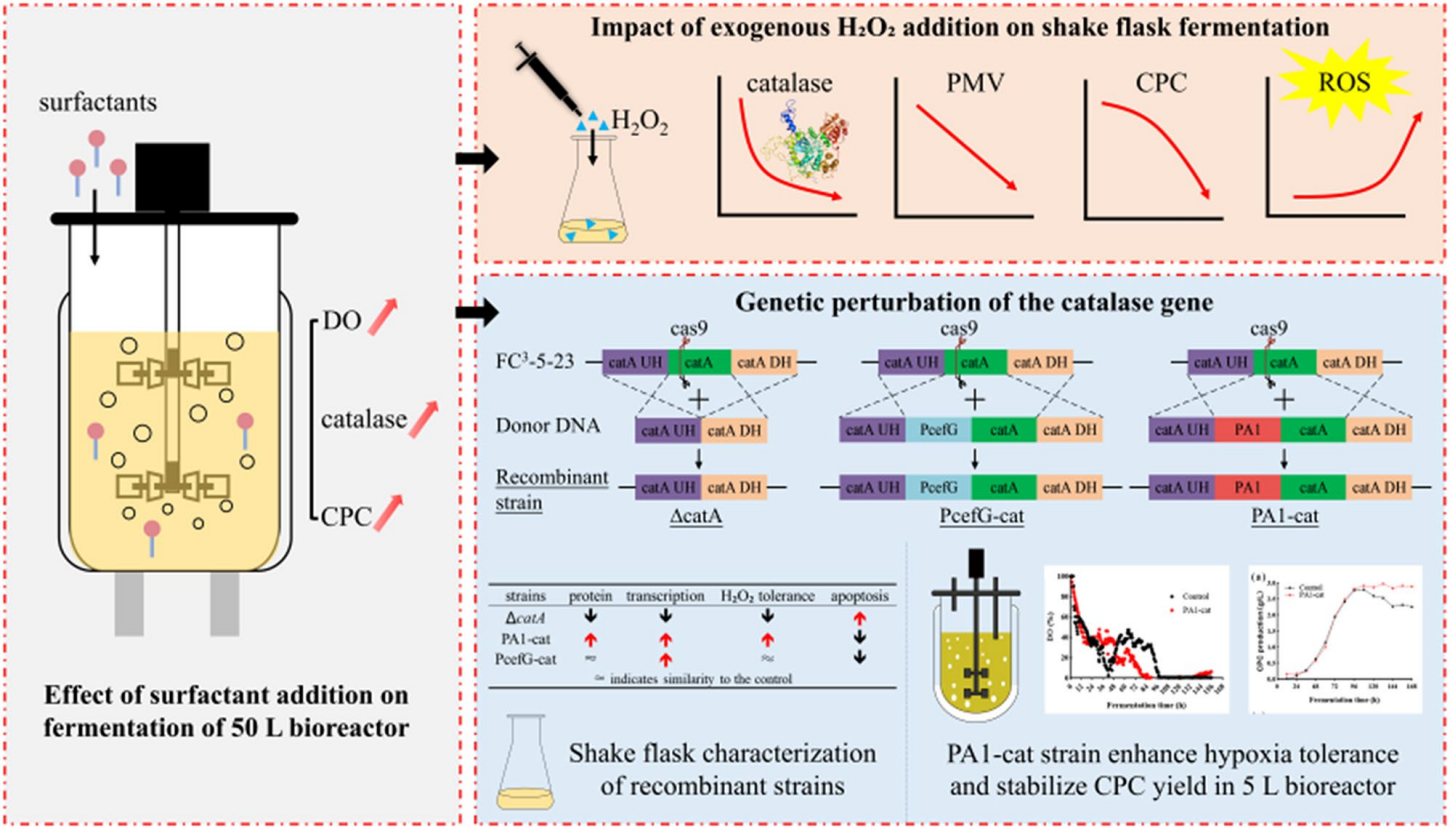

**Supplementary Information:**

The online version contains supplementary material available at 10.1186/s40643-024-00831-y.

## Introduction

Cephalosporin antibiotics are characterized by their broad antimicrobial spectrum, robust safety profiles, stability against β-lactamases, and minimal allergic reactions. Cephalosporin C is an essential raw material for synthesizing cephalosporin antibiotics; the 7-aminocephalosporanic acid (7-ACA) obtained by removing its branched chain serves as a starting point for the synthesis of many cephalosporins. The efficient production of CPC relies on an adequate oxygen supply, which is essential for both cellular energy metabolism (Molnár et al. 2018) and CPC biosynthesis (Zhou et al. 1992) during the fermentation of *A. chrysogenum*. Dissolved oxygen (DO), a key parameter reflecting the level of oxygen supply and consumption in the fermentation broth, serves as a crucial indicator for controlling fermentation processes (Kozma and Karaffa. 1996). Adjusting aeration (Venugopal et al. 2007) or agitation (Bakri et al. 2011), adding exogenous particles (Gonciarz et al. 2016), and regulating fermentation pressure (Man et al. 2016) are common approaches used to control DO level, thus regulating the cellular metabolism of filamentous fungi (Songserm et al. 2018; Zhang et al. 2012). Additionally, the heterologous expression of the *Vitreoscilla* hemoglobin gene (*vgb*) in *A. chrysogenum* has been reported to increase cellular affinity for oxygen, thereby enhancing CPC production (DeModena et al. 1993). Yang et al. designed a novel impeller combination using computational fluid dynamics (CFD) simulation, effectively enhancing oxygen mass transfer during fermentation and significantly improving CPC production (Yang et al. 2012). Surfactants, a class of organic compounds with both hydrophilic and hydrophobic properties, reduce the surface tension of liquids, thereby facilitating the entry of oxygen into cells during fermentation (Jamnongwong et al. 2010). Surfactants have proven effective in enhancing the biosynthesis of secondary metabolites, such as increasing the production of kitasamycin in *Streptomyces kitasatoensis* (Zheng and Gao. 2016) and echinocandin B in *Aspergillus nidulans* (Niu et al. 2021).

The use of oxygen as a terminal electron acceptor in the cellular respiratory chain frequently coincides with oxidative stress, primarily caused by reactive oxygen species (ROS) (Li et al. 2009), which include highly reactive oxygen-containing molecules such as superoxide anions (O₂⁻), H₂O₂, and hydroxyl radicals (OH⁻). ROS regulate cellular differentiation and secondary metabolite biosynthesis in fungi (Scott and Eaton. 2008). Reports have indicated that an appropriate increase in ROS levels facilitates conidia formation and aflatoxin biosynthesis in *Aspergillus flavus* (Zhu et al. 2020). A similar outcome has been observed in CPC biosynthesis; however, excessive ROS accumulation leads to cell apoptosis (Bibián et al. 2020). To counteract cellular damage caused by ROS accumulation, cells generate antioxidant enzymes and utilize antioxidant systems to maintain homeostasis (Belozerskaya and Gessler. 2007; Yaakoub et al. 2022). Enzymatic antioxidant systems, including superoxide dismutase (Kobayashi et al. 2019; Wang et al. 2018), catalase (Pradhan et al. 2017), glutathione peroxidase (Zhang et al. 2020), and the thioredoxin system (Ma et al. 2018), play crucial roles in maintaining intracellular ROS levels (Staerck et al. 2017). Specifically, superoxide dismutase targets superoxide radicals, while glutathione peroxidase, catalase, and thioredoxin systems primarily eliminate intracellular H₂O₂. Knocking out the sulfhydryl reductase-encoding gene *ActrxR1* in the thioredoxin system of *A. chrysogenum* CGMCC 3.3795 doubled CPC production, possibly due to the stimulation of CPC biosynthesis by increased ROS (Liu et al. 2013). Disruption of the glutathione reductase-encoding gene *glrA* in the same strain resulted in decreased cellular growth, CPC production, and antioxidant capacity. Therefore, it is assumed that the *glrA* gene functions to resist high-intensity oxidative stress, while the *ActrxR1* gene may specifically counteract low-intensity oxidative stress (Long et al. 2012). However, relevant studies on the role of catalase in cell growth and apoptosis, ROS levels, and CPC biosynthesis in *A. chrysogenum* are still unclear.

In this study, the effects of surfactant addition on cellular oxygen metabolism, catalase biosynthesis, and CPC production during fermentation were initially examined. Subsequently, the physiological roles of catalase and its effects on CPC biosynthesis were further investigated through the exogenous addition of H₂O₂ and genetic perturbation of the catalase encoding gene *catA*.

## Materials and methods

### Strains and plasmids

The strains and plasmids used in this study are listed in Table 1.

**Table 1:** Strains and plasmids used in this study

Strains or plasmids	Characteristics	Source
Strains		
DH5α	*E. coli* was used for cloning and propagation	Our lab
FC^3^-5-23	* A. chrysogenum* industrial cephalosporin C producing strain	Our lab
Ac-∆axl2::egfp	*Acaxl2* deleted in FC^3^-5-23, open reading frame was substituted by donor DNA *egfp*, promoted by P*gpdA*	Our lab
∆*catA*	FC^3^-5-23 Strain with ∆*catA*	This work
PA1-cat	∆*catA* and overexpression of *catA* under strong promoter P_A1_	This work
PcefG-cat	∆*catA* and overexpression of *catA* under weak promoter P_*cefG*_	This work
Plasmids		
pFC332	Replicated autonomously plasmid with *Aspergillus* optimized Cas9 and *hph* selection marker	Addgene
pFC332-∆cat	pFC332 inserted with sgRNA (*catA*)	This work
pMD18-T	commonly used plasmid vector for TA-cloning	Takara
T-PA1-cat	pMD18-T-cat UDH-PA1-catA	this work
T-PcefG-cat	pMD18-T-cat UDH-PcefG-catA	This work

### Plasmid construction process

The primers used in this study are listed in Table 2. The construction of plasmid pFC332-∆cat followed the same steps as those for pFC332-∆target (Liu et al. 2022). The plasmid construction processes for T-PA1-cat and T-PcefG-cat are illustrated in Fig. S1. Initially, cat-UH-F/R and cat-DH-F/R were used as primers to amplify the left and right homologous arms of *catA* from FC^3^-5-23 genomic DNA, respectively. After fusing the left and right homologous arms of *catA*, the fused fragment was ligated into a T-vector, named T-cat UDH. The long fragments were reverse-amplified using the inverse-trpC-F and inverse-PA1-R primers. Additionally, the short fragments were amplified using cat-F and cat-his-R primers from FC^3^-5-23 genomic DNA. After ligating these two fragments using a one-step cloning kit (purchased from Vazyme, Nanjing, China), an intermediary plasmid was obtained, named T-sorB UDH-PA1-catA. Using T-cat UDH as a template, long fragments were reverse-amplified with inverse-cat UDH-F/R primers. Meanwhile, short fragments were amplified using PA1-cat-F and cat-trpC-R primers with T-sorB UDH-PA1-catA as a template. These two fragments were ligated using a one-step cloning kit, which resulted in the final desired plasmid, T-PA1-cat. The construction process for T-PcefG-cat follows the same steps as described above. The final constructed plasmids are utilized in subsequent PCR to obtain the homologous recombination donor DNA fragments, which are generated through PCR amplification using the primers cat-UH-F and cat-DH-R.

**Table 2:** Primers used in this study

Primer Name	Sequence(5’-3’)
cat-UH-F	gaaccgtccgtcacgactcacacatagctt
cat-UH-R	ttgggtggacgcattgaagaaagtgccccggggatgttttag
cat-DH-F	ctaaaacatccccggggcacttaggggccaagggcgctggggtgggcgc
cat-DH-R	cgactccttcgtctgggccgtcgtcaacaagac
Inverse-cat UDH-F	tgaaggggccaagggcgctggg
Inverse-cat UDH-R	aagtgccccggggatgttttag
Inverse-trpC-F	catcatcatcatcaccactgaggatccacttaacgttactgaaatc
Inverse-PA1-R	cggctagtaccgcggaacgaatcatctttgcgactttcgctaaaggtggg
Inverse-PcefG-R	cggctagtaccgcggaacgaatcatggtgggcgacgtgtggctgtaggtg
cat-F	atgattcgttccgcggtactagccgctac
cat-his-R	tcagtggtgatgatgatgatgcgccccatcaagcgcaaacct
PA1-cat-F	ctaaaacatccccggggcactttgcgctggtgtggtctgaagggta
PcefG-cat-F	ctaaaacatccccggggcacttgttgatgctgtggttttgagcg
cat-trpC-R	cccagcgcccttggccccttcatcgagtggagatgtggagtggg

### Cultivation protoplast transformation and verification in *A. chrysogenum*

The storage, cultivation, and fermentation processes for FC^3^-5-23 and Ac-∆axl2::egfp were described previously (Xu et al. 2022). The formulation of the 50 L bioreactor fermentation medium was formulated based on Li et al. (2010), with 20 g of soybean powder and 10 g of wheat gluten added. The genetic manipulation platform established for CGMCC 3.3795 (Liu et al. 2022) was confirmed to be effective in FC^3^-5-23. The surfactants, referred to as the RD series, were provided by BASF (China) Co., Ltd. for this study. These surfactants are not commercially available and have not been previously reported in the literature. According to BASF, the RD series consists of polymeric polyether surfactants synthesized by copolymerizing ethylene oxide (EO) and propylene oxide (PO). The polyoxyethylene portion of the molecule is hydrophilic, whereas the polyoxypropylene segment is hydrophobic. Their performance varies based on the relative molecular mass and the EO/PO ratio. Specifically, within the RD100-103 range, the proportion of PO remains constant while the EO content gradually increases; in contrast, in the RD107-109 range, the EO content is fixed while the PO content increases.

After centrifuging the culture samples at 4000 g for 10 min at 4 °C, the packed mycelium volume (PMV) was calculated as the ratio of the settled mycelium volume to total culture volume to assess cell growth (Cai et al. 2024; Wu et al. 2021).

Transfer 10 mL of the fermentation broth into a pre-weighed centrifuge tube labeled “a”. Centrifuge, wash the pellet twice with sterile water, and centrifuge it again. Add diluted hydrochloric acid to hydrolyze any remaining calcium carbonate. Centrifuge to remove the supernatant, then open the centrifuge tube, and dry it at 65 °C in an oven. Weigh the tube after drying, denoting it as “b”. Calculate the dry cell weight (DCW) using the formula: DCW = (b - a) / 10 (mg/mL).

### Fermentation parameters and CPC measurement

The 5 L and 50 L bioreactors were purchased from Shanghai Guoqiang Bioengineering Equipment Co., Ltd.(Shanghai, China). The media and cultivation conditions for the bioreactors were supplied by Shanxi Weiqida Pharmaceutical Co., Ltd. The oxygen uptake rate (OUR) and carbon dioxide evolution rate (CER) were determined using a process mass spectrometer (MAX300-LG, Extrel, USA), as described previously (Li et al. 2022). The DO in broth was measured using an oxygen electrode (Mettler Toledo). The CPC production was quantified using high-performance liquid chromatography (HPLC), as previously described (Xu et al. 2022).

### SDS-PAGE and Western blot

The Omni-Easy™ One-Step PAGE Gel Fast Preparation Kit (PG213) was used to prepare the gels according to the provided instructions. Extracellular proteins were isolated from the supernatant of the centrifuged fermentation broth after appropriate dilution. Intracellular proteins were extracted from lysed cultured cells. Both samples were combined with 5X loading buffer, boiled for 5–10 min, cooled to room temperature, and then subjected to SDS-PAGE. Subsequently, the gels were stained with Coomassie blue, decolorized, and imaged for further analysis. The Western blotting method described in the general protocol provided by Bio-Rad was slightly modified for the purposes of this study.

### Measurement of intracellular and extracellular protein content

A 1 mL sample of fermentation broth was collected, and the clarified supernatant obtained through centrifugation was used as the extracellular protein sample. The mycelium layer was washed 3–4 times with phosphate-buffered saline (PBS) until the supernatant became clear. The mycelium was then suspended in 1 mL of PBS containing glass beads and 10 µL of 100 mM phenylmethylsulfonyl fluoride (PMSF). The resulting mixture was frozen, ground, and centrifuged to obtain a cell-free extract, which served as the intracellular protein sample. The protein content was measured using the Omni-Easy™ Instant BCA Protein Assay Kit (ZJ102) (Yan et al. 2021). To meet the detection range, the protein samples were diluted.

### Catalase activity assay

Catalase activity was measured according to the instructions provided in the Catalase Assay Kit (G0105W) from Suzhou Gracell Biotechnologies Co., Ltd. Catalase activity was defined as one unit (U), which catalyzes the decomposition of 1 µmol of H₂O₂ per minute per milligram of intracellular protein at 25 °C.

### ROS measurement

Firstly, 500 µL of the cell-free extract was mixed with 2.5 µL of 2 mM 2,7-dichlorodihydrofluorescein diacetate (DCFH-DA) dissolved in ethanol and incubated in the dark at room temperature with shaking for 30 min. Subsequently, 200 µL of the reaction mixture was measured using the Varioskan LUX multimode microplate reader (Thermo, Waltham, USA) at an excitation wavelength of 485 nm and an emission wavelength of 522 nm.

### H₂O₂ sensitivity assay

To assess the sensitivity of the strains to H₂O₂, a solid plate cultivation method previously described in the literature was utilized (Xu et al. 2022). MM solid medium supplemented with the appropriate volume of H₂O₂ was prepared to evaluate the tolerance of recombinant strains to H₂O₂.

### Cell apoptosis detection

Firstly, 1 mL of fermentation broth was centrifuged and washed twice with PBS. The pellet was resuspended in 1 mL of PBS, and 10 µL of 10 µg/mL propidium iodide (PI) solution was added, followed by incubation at 37 °C for 1 h. While PI cannot penetrate intact cell membranes, it can enter cells through damaged membranes and stain the nucleus, thereby effectively assessing apoptosis (Kari et al. 2022). After incubation, the cells were washed twice with PBS and resuspended in 1 mL of PBS. Then, 200 µL of the suspension was measured using a microplate reader at an excitation wavelength of 535 nm and an emission wavelength of 615 nm.

### Statistical analysis

The data are presented as the mean ± standard deviation (s.d.) from three independent biological experiments. Statistical significance levels are indicated by asterisks: * for *p* < 0.05, ** for *p* < 0.01, *** for *p* < 0.001, and **** for *p* < 0.0001. “ns” indicates no significance.

## Results

### Effects of surfactants on CPC fermentation by *A. chrysogenum*

In preliminary experiments, we examined the effects of adding surfactants at various times and concentrations in shake flask cultures (Fig. S2). The results showed that adding 1% surfactant at 72 h did not significantly increase CPC yield compared to FC^3^-5-23 fermentation without surfactant. However, adding 0.3% surfactants (RD100, RD102, and RD103) at 60 h significantly enhanced CPC yield (Fig. S2c). Additionally, apart from RD101, none of the other surfactants had a significant impact on PMV compared to the control (Fig. S2d).

**Fig. 1:**
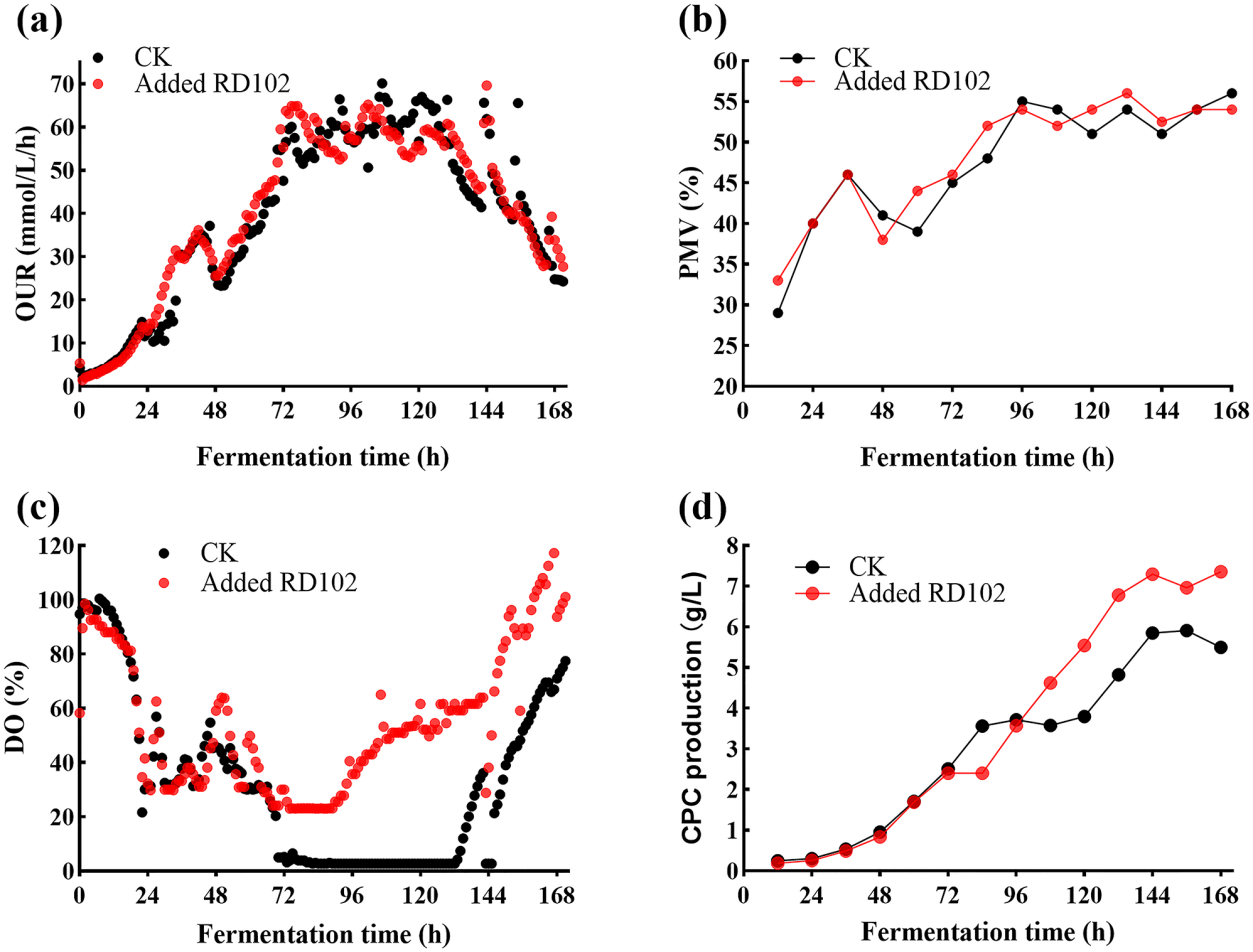
The effect of surfactant RD102 on the CPC fermentation of FC^3^-5-23 in a 50 L bioreactor. “CK” refers to the control group with the FC^3^-5-23 strain without surfactant addition, while “Added RD102” represents the group with surfactant RD102 addition. RD102 is a polymer polyether surfactant provided by BASF. (**a**) OUR, Oxygen uptake rate, reflects the rate of oxygen consumption during fermentation; (**b**) PMV, Packed mycelium volume, reflects the content of mycelium in the fermentation broth; (**c**) DO, Dissolved oxygen; (**d**) CPC production

We selected RD102 to further investigate the effects of surfactant addition on CPC fermentation by the FC^3^-5-23 strain in the 50 L bioreactor. Cell growth in the 50 L bioreactor was superior to that in the shake flask cultures. Therefore, RD102 was added at 48 h with a consistent dosage of 0.3%, to assess its impact on FC^3^-5-23 fermentation in the 50 L bioreactor (Fig. 1). No significant differences in OUR or PMV were observed between the experimental and control groups (Fig. 1a-b), indicating that RD102 did not significantly affect cell growth. The higher PMV observed in the 50 L bioreactor fermentation, compared to the shake flasks, can be attributed to several factors: superior mass transfer and DO conditions, richer media composition, and controlled environmental parameters that closely resemble industrial production environments. The DO level in the control group consistently remained at zero between 70 and 130 h (Fig. 1c). During this phase, CPC production plateaued, indicating that the low DO level in the 50 L bioreactor had become a critical constraint, hindering continuous CPC production (Fig. 1d). Compared to the control group, the addition of RD102 significantly alleviated the drop in DO level to zero. Surfactants raise DO level by reducing surface tension, increasing solubility, enhancing interfacial activity, and improving liquid permeability (Hamzah et al. 2018). The surfactant interacts with soybean oil in the medium to promote emulsification, reduce the surface tension of the broth and the interfacial tension between oil and water, significantly lowering resistance to gas exchange (De et al. 2015). This improvement enhances oxygen mass transfer, creating a more favorable microenvironment for cells near the improved gas exchange area. Consequently, effective broth mixing further optimizes the overall fermentation environment.

### Extracellular protein expression under surfactant addition and catalase identification

**Fig. 2:**
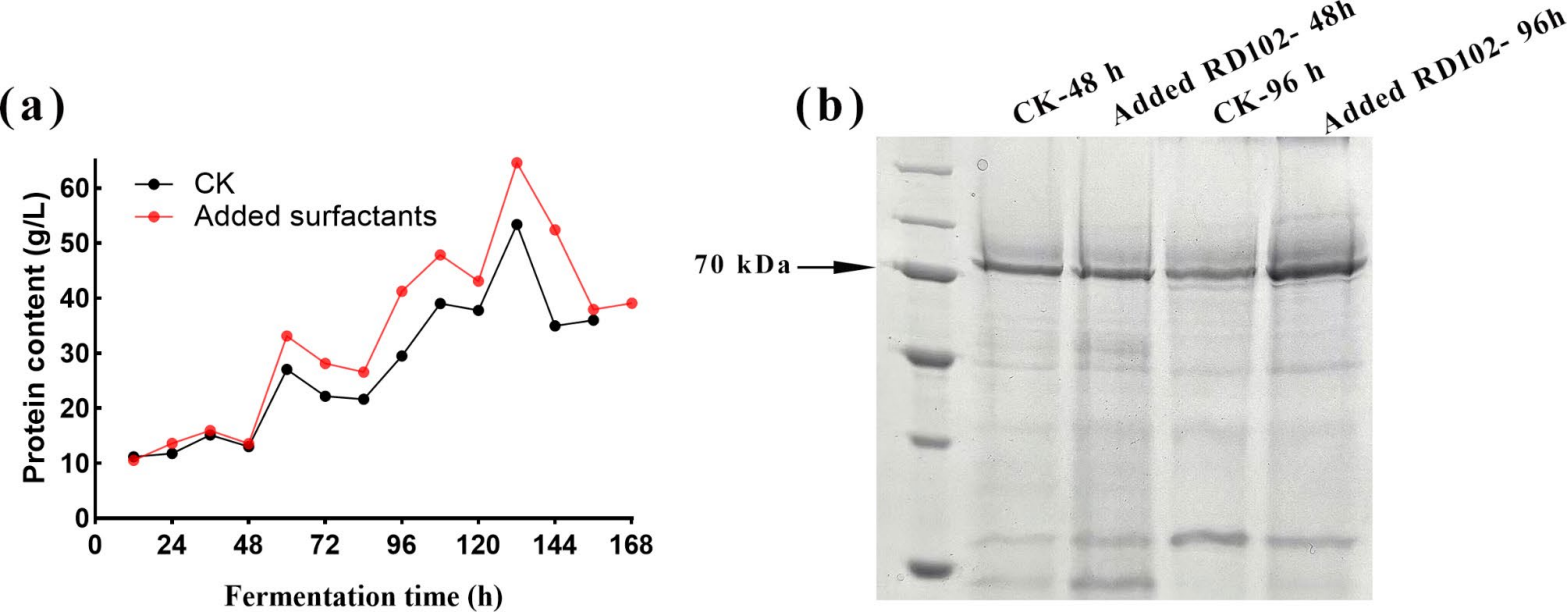
The effect of surfactant RD102 (a polymer polyether surfactant) on the CPC fermentation of FC^3^-5-23 in a 50 L bioreactor. “CK” refers to the control group with the FC^3^-5-23 strain without surfactant addition, “Added surfactants” and “Added RD102” represent the group with surfactant RD102 addition. (**a**) Comparison curve of extracellular total protein content; (**b**) SDS-PAGE results of extracellular proteins at 48 h and 96 h, with distinct bands observed above 70 kDa

The addition of RD102 to the 50 L bioreactor led to an increase in extracellular protein content (Fig. 2a). We hypothesized that the addition of surfactants might enhance the protein secretion capacity in FC^3^-5-23. SDS-PAGE analysis of extracellular proteins at 48 h and 96 h in both the experimental and control groups revealed that the experimental group exhibited a notable increase in secretion of an unidentified protein larger than 70 kDa (Fig. 2b).

To investigate the potential correlation between the unidentified protein and CPC production, mass spectrometry was employed to identify this protein and analyze its sequence and function. The mass spectrometry results revealed 160 peptide fragments and 30 protein species (Tables S1 and S2). The protein with the highest similarity was identified as catalase, with a molecular weight of 78.414 kDa (homologous to KFH49103.1 from ATCC 11550). As an antioxidant, catalase neutralizes intracellular ROS and promotes cell growth and secondary metabolism.

### Impact of exogenous H₂O₂ addition on shake flask fermentation of *A. chrysogenum*

**Fig. 3:**
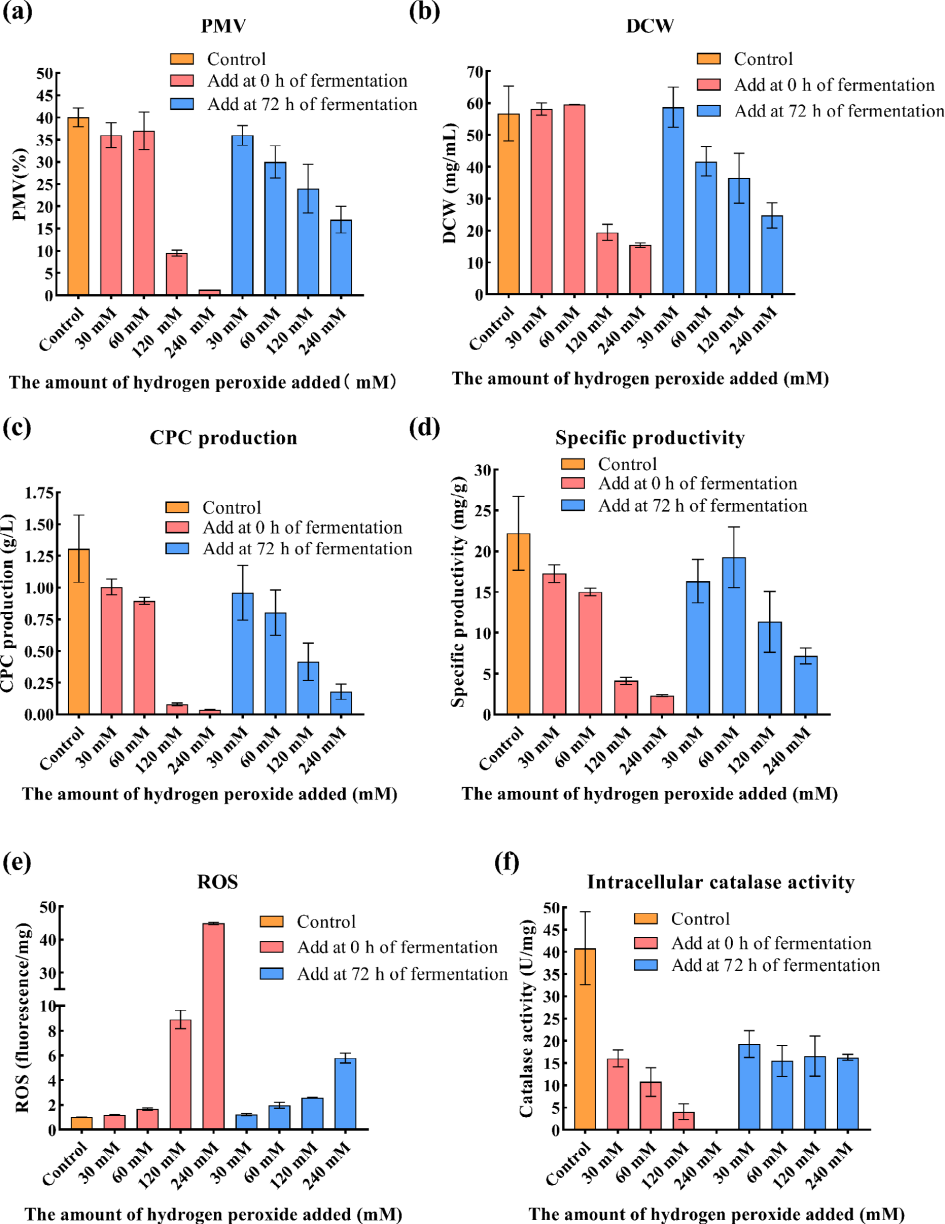
The effect of H₂O₂ addition on the shake flask fermentation of *A. chrysogenum* FC^3^-5-23. “Control” indicates the FC^3^-5-23 strain without H₂O₂ addition; “Add at 0 h of fermentation” refers to the addition of varying H₂O₂ concentrations at the start of fermentation; while “Add at 72 h of fermentation” refers to H₂O₂ addition at 72 h. (**a**) PMV, Packed mycelium volume; (**b**) DCW, Dry cell weight; (**c**) CPC production; (**d**) Specific productivity refers to the CPC yield per unit dry cell weight, calculated using the formula: Specific productivity(mg/g) = CPC production (g/L) *1000 / DCW(mg/mL); (**e**) ROS, normalized by unit mg DCW; (f) Intracellular catalase activity, normalized by unit mg intracellular protein content. All the results presented above were measured after a 7 day incubation and fermentation period

Moderate exogenous H₂O₂ addition has been shown to influence both ROS level and catalase activity, which have beneficial effects on aflatoxin production by *Aspergillus flavus* (Zhu et al. 2020), penicillin production by *Penicillium chrysogenum* Wisconsin 54-1255, and CPC production by wild-type strain *A. chrysogenum* ATCC 11,550 (Bibián et al. 2020). To further investigate the relationship between catalase level and CPC production, different concentrations of H₂O₂ were added at 0 h and 72 h during shake flask fermentation of FC^3^-5-23 (Fig. 3).

The results from shake flask cultivation showed that the addition of 30 mM and 60 mM H₂O₂ at 0 h resulted in a slight decrease in PMV, CPC production and specific productivity (Fig. 3a-d). In contrast, adding 120 mM and 240 mM H₂O₂ significantly inhibited cell growth, leading to a marked decline in both CPC production and specific productivity. Similarly, the addition of H₂O₂ at 72 h resulted in a decrease in PMV, DCW, CPC yield, and specific productivity. The addition of 30 mM, 60 mM, 120 mM, and 240 mM H₂O₂ at 0 h led to a decrease in CPC titers by 23.14%, 31.43%, 93.86% and 97.24%, respectively, compared to the control for FC^3^-5-23 (1.31 g/L). Meanwhile, the addition at 72 h decreased CPC titers by 26.56%, 38.51%, 68.28%, and 86.36%, respectively. A clear inverse relationship was observed between CPC yield and increasing H₂O₂ concentration, likely due to intracellular ROS perturbation induced by the exogenous addition of H₂O₂.

This speculation was confirmed by the results of the ROS assay during shake flask cultivation (Fig. 3e), which demonstrated a significant increase in intracellular ROS level following the addition of exogenous H₂O₂. The addition of 30 mM, 60 mM, 120 mM, and 240 mM H₂O₂ at 0 h increased ROS level by 0.197-fold, 0.627-fold, 7.77-fold, and 42.39-fold, respectively, compared to the control group. Meanwhile, the addition at 72 h increased ROS level by 0.218-fold, 0.94-fold, 1.54-fold, and 4.70-fold, respectively. The addition of H₂O₂ at 0 h resulted in a more pronounced accumulation of intracellular ROS in FC^3^-5-23 compared to the addition at 72 h. This suggests that *A. chrysogenum* FC^3^-5-23, having progressed past the growth retardation phase and into the late growth phase by 72 h, possesses enhanced tolerance and equilibrium capacity for exogenous H₂O₂. This observation aligns with the high expression and secretion of catalase protein observed in this strain after 48 h in a 50 L bioreactor (Fig. 2b).

Furthermore, the addition of exogenous H₂O₂ significantly reduced catalase activity of the FC^3^-5-23 strain (Fig. 3f). When H₂O₂ was added at 0 h, catalase activity decreased dramatically with increasing H₂O₂ concentration. Similarly, when H₂O₂ was added at 72 h, catalase activity was significantly lower than in the control group without H₂O₂. However, unlike the addition at 0 h, catalase activity in the 72 h addition group remained relatively stable, with only a slight and indistinct decrease as the H₂O₂ concentration increased. Based on the comparative results between the 0 h and 72 h additions, we propose that the FC^3^-5-23 strain, having entered the late growth stage (i.e., under the 72 h addition condition), may exhibit a stronger intracellular redox balance and higher H₂O₂ tolerance than the original strain at 0 h. The secretion of catalase exhibited a decreasing trend as the level of H₂O₂ addition increased. Additionally, the secretion of extracellular proteins by the FC^3^-5-23 strain is shown under various conditions of H₂O₂ addition (Fig. S3). Based on semi-quantitative protein analysis, the estimated catalase protein content relative to the total protein is depicted in Fig. S4.

Overall, the addition of exogenous H₂O₂ not only significantly increased intracellular ROS level and impeded cell growth but also significantly decreased both intra- and extracellular catalase activity and content, adversely affecting CPC fermentation in *A. chrysogenum*. A correlation was observed between the decrease in CPC production and the decline in catalase activity. Higher CPC production appeared to coincide with increased catalase activity, consistent with the comparative catalase activity observed between CGMCC 3.3795 and Ac-∆axl2::eGFP (Fig. S5). The transcription level of the *catA*, extracellular catalase protein content, and intracellular catalase activity of the high-yielding CPC strain were significantly higher than those of the wild-type strain CGMCC 3.3795. The relationship between catalase and CPC production was subsequently examined in greater detail.

### Physiological function analysis of *catA* gene

Characterization of catalase secretion, enzyme activity, and H_2_O_2_ tolerance in three *catA* recombinant strains during shake flask cultures.

**Fig. 4:**
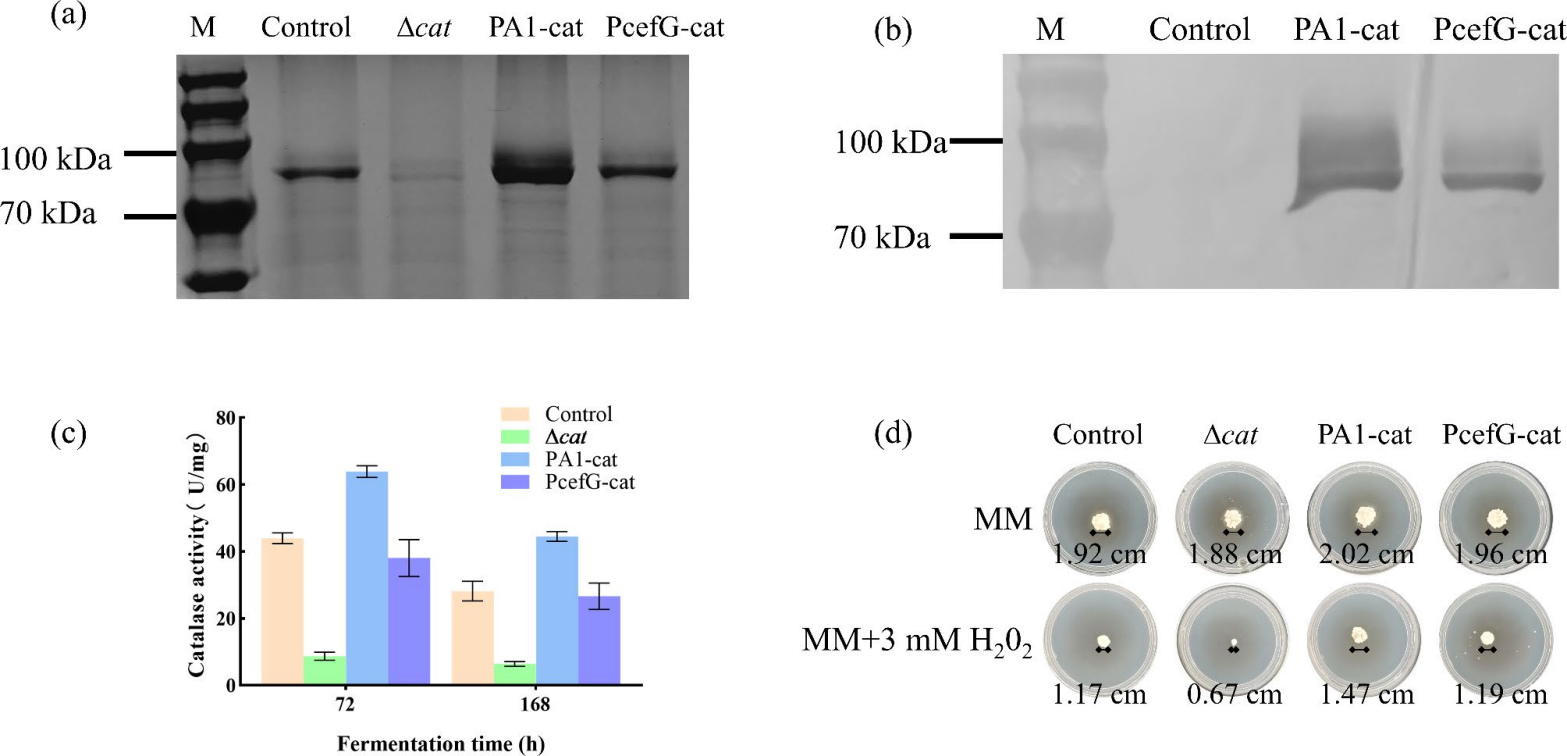
Functional characterization of the *catA* recombinant strains at the shake flask scale. “Control” refers to the FC^3^-5-23 strain, and “∆cat” refers to the knock-out of *catA* gene in FC^3^-5-23 strain; “PA1-cat” denotes the FC^3^-5-23 strain modified to express *catA* under the strong promoter PA1; “PcefG-cat” represents the FC^3^-5-23 strain modified to express *catA* under the weak promoter PcefG. Details on strain construction and characteristics are provided in Supplementary Fig. S1 and Table 1 (**a**) SDA-PAGE analysis of extracellular proteins; (**b**) Western blot of extracellular proteins; (**c**) Comparison of catalase activity at 72 h and 168 h; (**d**) Comparison of the growth of different recombinant strains on solid minimal medium and plates with additional H₂O₂. Colony diameter values indicate growth after a fixed incubation period

To further elucidate the relevance of catalase and its encoding gene *catA* to cell growth and CPC biosynthesis, the expression level of *catA* in the FC^3^-5-23 strain was modified using a strong promoter (PA1), a weak promoter (PcefG), and *catA* knockout. The overexpressed *catA* gene sequences were fused with His-tags to facilitate subsequent Western blot experiments for the identification of catalase proteins. The *catA* recombinant strains exhibited comparable alterations at both the transcriptional and protein levels (Fig. 4). Specifically, compared to FC^3^-5-23, the ∆*catA* strain ceased catalase secretion, resulting in a significant reduction in intracellular catalase activity (Fig. 4a, c). The overexpressing strain, PA1-cat, exhibited higher extracellular protein levels compared to FC^3^-5-23 (Fig. 4a-b). At 72 h and 168 h, the intracellular catalase activities in the overexpressing strain PA1-cat were 1.44 times and 1.43 times higher, respectively, compared to FC^3^-5-23 (Fig. 4c). The weak expression strain, PcefG-cat, did not show significant differences in specific enzyme activity at different time points compared to FC^3^-5-23. The cell growth of the *catA* recombinant strains in minimal medium (MM) was comparable to that of the control strain (Fig. 4d). In media containing 3 mM H₂O₂, the ∆*catA* strain was more sensitive to H₂O₂, consistent with expectations, displaying smaller colonies than those of the control strain. The PA1-cat strain exhibited increased H₂O₂ tolerance, whereas the sensitivity of the PcefG-cat strain to H₂O₂ was similar to that of FC^3^-5-23 (Fig. 4d).

### Shake flask fermentation of *catA* recombinant strains

**Fig. 5:**
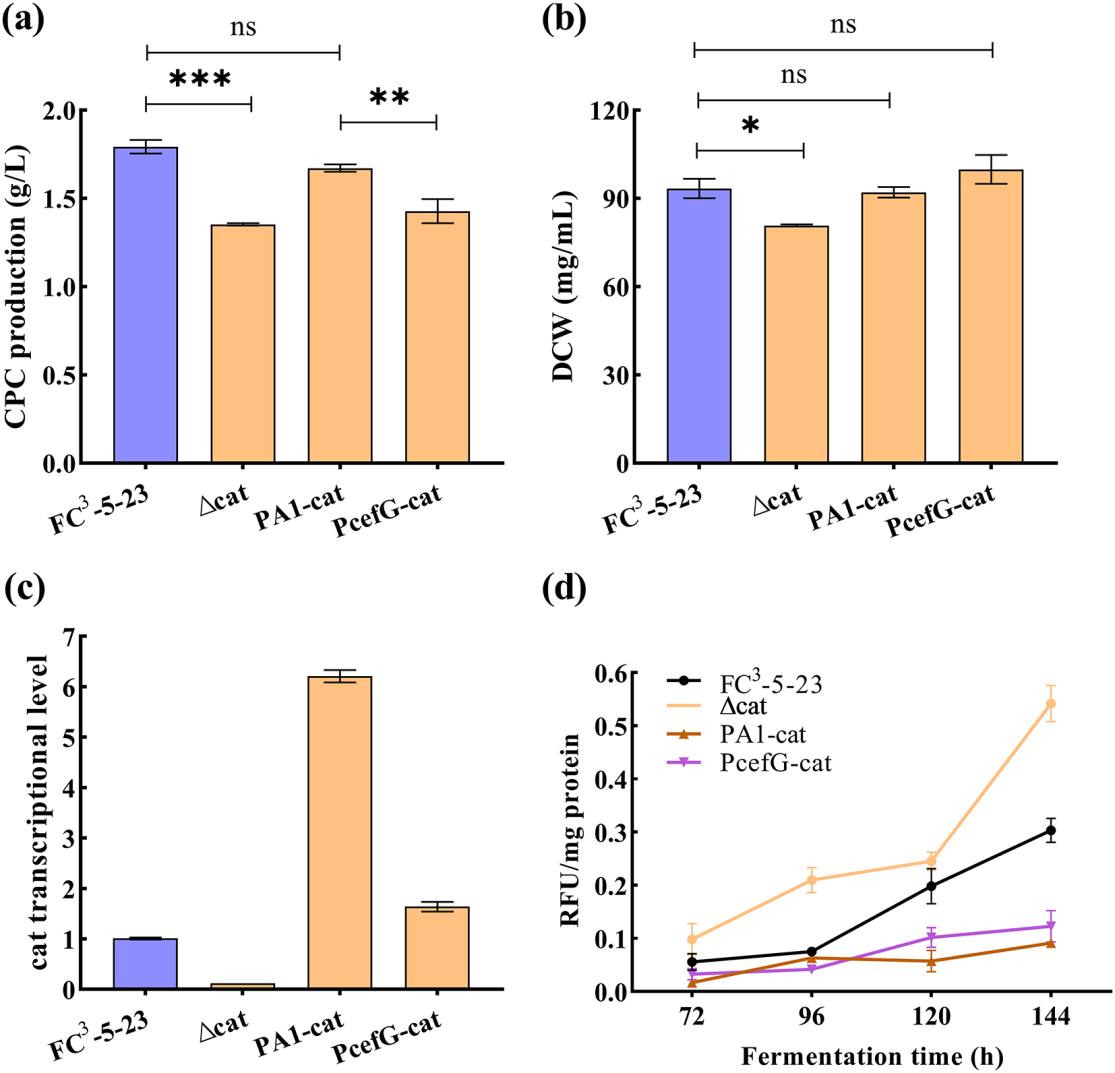
Functional identification of *catA* recombinant strains at the shake flask scale.* for *p* < 0.05, ** for *p* < 0.01, *** for *p* < 0.001 in the figure. “ns” means no significance. (**a**) CPC production; (**b**) DCW, Dry cell weight; (**c**) *catA* transcriptional level; (**d**) Apoptosis curves at different fermentation time. Apoptosis was assessed using fluorescence detection following staining with propidium iodide (PI). Higher fluorescence values indicate a greater number of apoptotic cells

The results showed significantly lower DCW and CPC yields of the ∆*catA* strain in shake flask cultivation compared to the control strain FC^3^-5-23 (Fig. 5a-b). The decline in DCW might be the primary reason for the decrease in CPC production of the ∆*catA* strain, as there was no significant difference in specific productivity between ∆*catA* and FC^3^-5-23. At the end of fermentation, the DCW of both PA1-cat and PcefG-cat did not differ significantly from that of FC^3^-5-23 (Fig. 5b). Compared to FC^3^-5-23, the transcriptional level of *catA* decreased by 86.88% in ∆*catA*, while increasing by 516% in PA1-cat and by 55.56% in PcefG-cat at the end of fermentation (Fig. 5c). Notably, at the end of fermentation, although the PA1-cat strain exhibited significantly increased catalase activity, this enhancement did not correspondingly influence CPC production (Figs. 4c and 5a). This observation suggests that the relationship between catalase activity and CPC production is not a straightforward positive correlation, potentially involving more complex metabolic regulatory mechanisms.

Additionally, further examination of the apoptosis process in the *catA* recombinant strains revealed an increase in apoptosis in all strains as fermentation time advanced (Fig. 5d). Notably, apoptosis in the ∆*catA* strain was significantly higher than that in FC^3^-5-23, while both overexpressing *catA* strains exhibited lower apoptosis compared to FC^3^-5-23 at the end of fermentation. This suggests that catalase, potentially by mitigating intracellular ROS-induced cellular damage, reduces the occurrence of apoptosis during late-stage fermentation.

### Differences in mycelial morphology during shake flask fermentation of *catA* recombinant strains

**Fig. 6:**
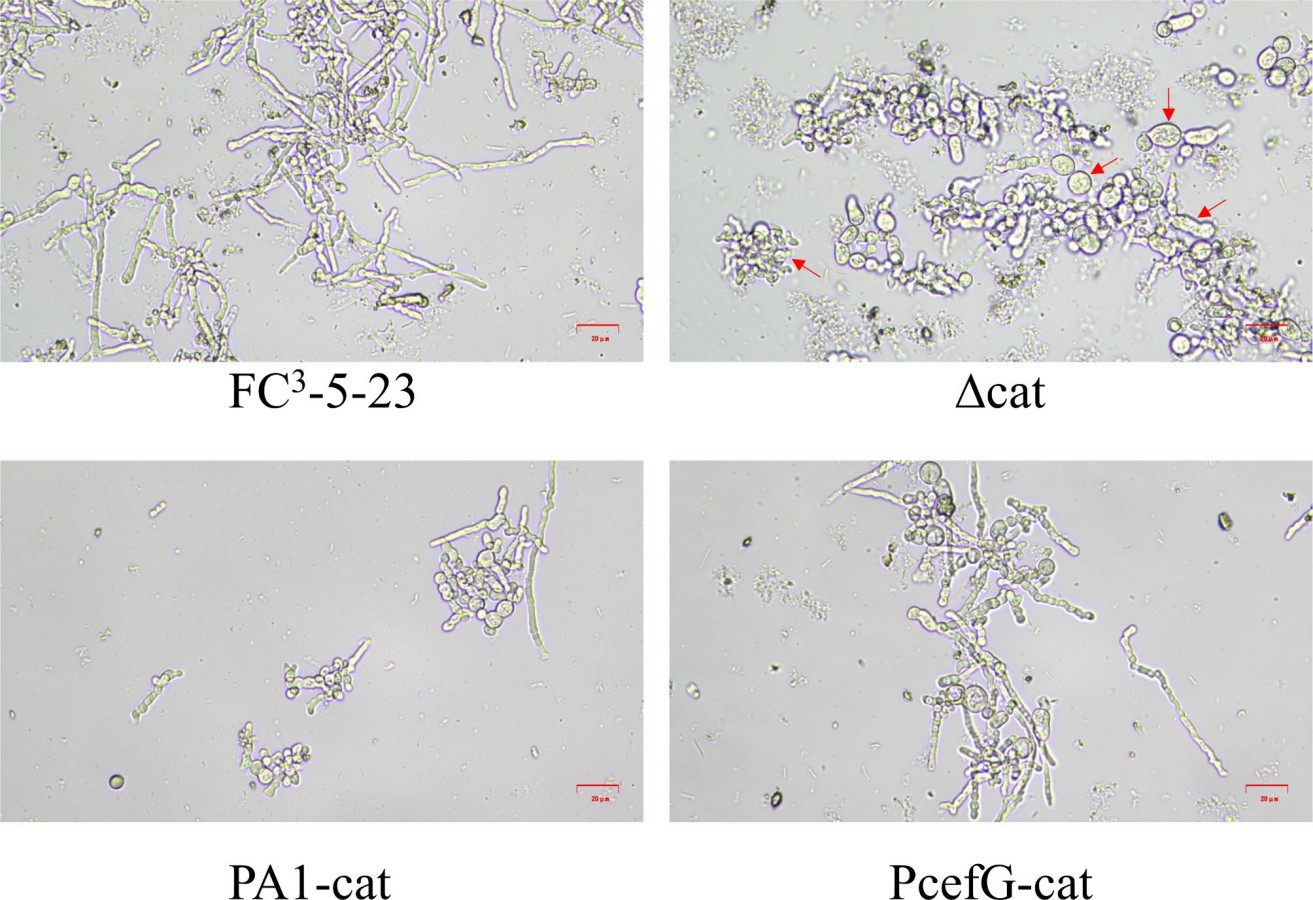
Morphology of hyphae and arthrospores in *catA* recombinant strains at 72 h of shake flask fermentation. Red arrows indicate hyphal fragment and larger swollen arthrospores. The scale bar represents 20 μm

At 72 h of fermentation, significant intracellular differentiation changes were observed among the different *catA* recombinant strains compared to FC^3^-5-23, especially characterized by the presence of hyphal fragments and larger swollen arthrospores in ∆*catA* strain (Fig. 6). This aberrant morphology may correlate with the reduced growth capacity, decreased CPC production, and increased apoptosis observed in ∆*catA* strain. Furthermore, both *catA* overexpressing strains exhibited more arthrospores than FC^3^-5-23, indicating that the overexpression of *catA* accelerated hyphal differentiation. The knockout of *cta1* in *Aspergillus flavus* has been shown to impact conidia formation (Zhu et al. 2020), indicating a widespread influence of catalase on cell growth and development in filamentous fungi.

### 5 L bioreactor fermentation of PA1-cat overexpression strain

**Fig. 7:**
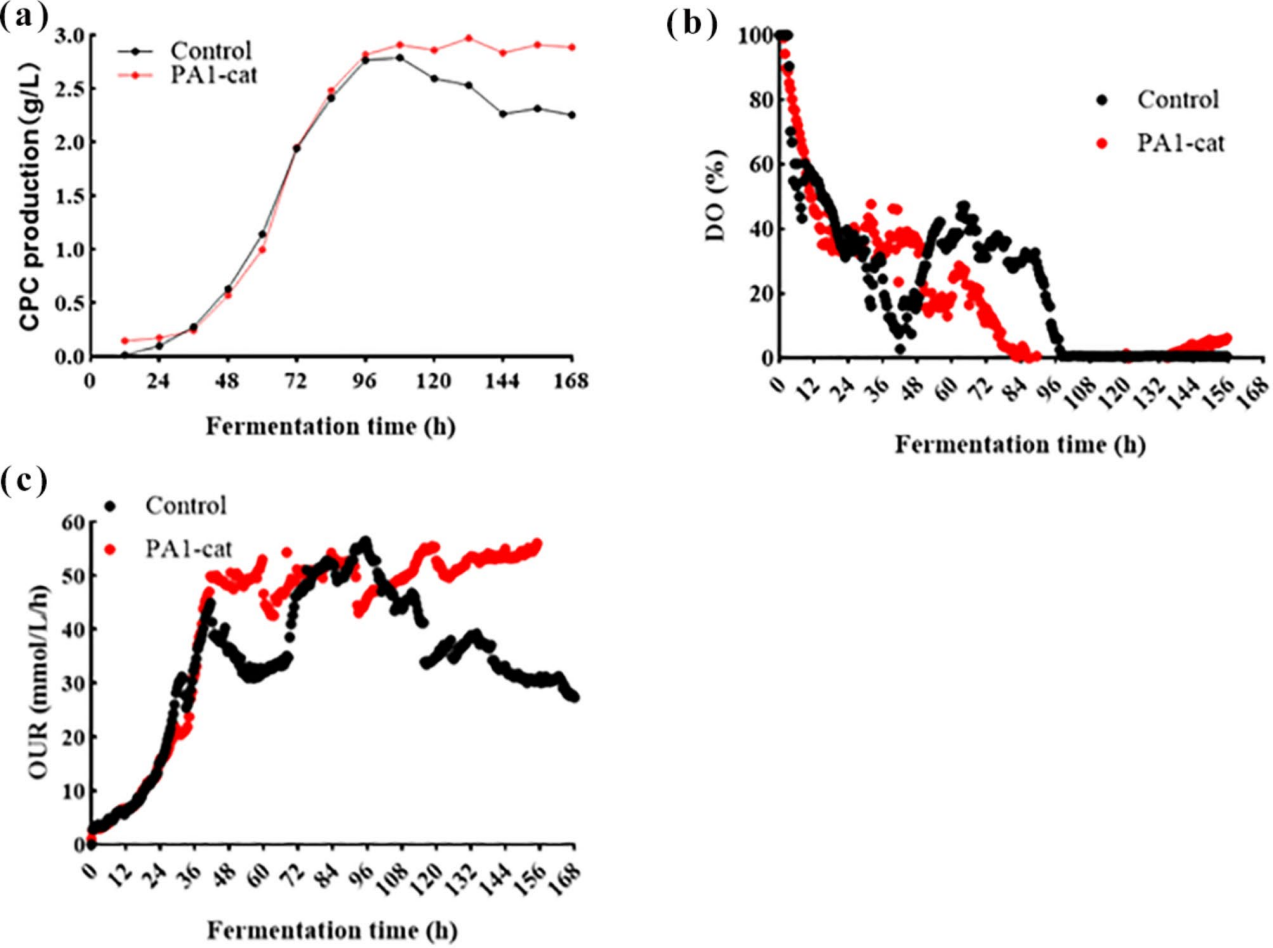
CPC fermentation of *A. chrysogenum* FC^3^-5-23 and PA1-cat strains in 5 L bioreactor. “PA1-cat” denotes the FC^3^-5-23 strain modified to express *catA* under the strong promoter PA1. (**a**) CPC production curve; (**b**) DO(Dissolved oxygen) variation curve; (**c**) Comparison of OUR(Oxygen uptake rate) curve during fermentation

The absence of baffles in the shake flasks, the use of atmospheric pressure fermentation, and the inability to accurately control factors such as DO and pH during the fermentation process may have resulted in relatively uncontrolled conditions that do not adequately reflect the overexpression of catalase in the PA1-cat strain. To further investigate the relationship between catalase, CPC production, and DO level, a 5 L batch fermentation experiment was conducted using strains FC^3^-5-23 and PA1-cat (Fig. 7). While PA1-cat did not significantly increase CPC production compared to FC^3^-5-23, it notably delayed the decline in CPC yield caused by insufficient oxygen supply, maintaining a higher production level during the later stages of fermentation (Fig. 7a). Unlike the addition of surfactants, which elevated the DO level, PA1-cat did not significantly affect DO in the 5 L bioreactor (Fig. 7b), indicating that surfactants were the primary factor driving DO enhancement in the 50 L bioreactor (Fig. 1). Despite the increased expression of the *catA* gene and elevated catalase activity in PA1-cat, the oxygen generated through enzymatic reactions was insufficient during the stable production phase. Furthermore, PA1-cat demonstrated greater stability in OUR (Fig. 7c), suggesting that the overexpression of *catA* enhances metabolic activity and improves adaptation and tolerance to oxygen-limited conditions.

Overall, the *catA* in *A. chrysogenum* plays a multifaceted role in regulating apoptosis, mycelial differentiation, and adaptation to hypoxic environments, in addition to managing oxidative stress. The significant overexpression of the *catA* enhances tolerance to oxidative stress, reduces apoptosis, improves adaptation to hypoxic conditions, and stabilizes both strain viability and CPC production under oxygen-limited environments.

## Discussion

Cephalosporin C (CPC) is a crucial precursor for cephalosporin antibiotics. Insufficient oxygen supply during fermentation is generally regarded as the primary factor limiting the efficient biosynthesis of CPC. In this study, we found that the addition of surfactants significantly enhanced the level of dissolved oxygen, the secretion of extracellular proteins, and the yield of CPC during fermentation. Notably, we unexpectedly identified a highly expressed protein among the secreted extracellular proteins, which appeared as a band above 70 kDa on SDS-PAGE. Analysis of the CPC biosynthetic gene cluster revealed no known genes encoding proteins of this molecular weight, leading us to speculate that this unknown protein might be involved in regulating CPC biosynthesis. Further analysis using liquid chromatography-mass spectrometry identified the protein as catalase.

Although catalase does not directly participate in the biosynthesis of CPC, it may indirectly influence CPC fermentation yield as a key enzyme in maintaining cellular redox homeostasis, either by regulating intracellular ROS level or by altering its own expression level. To validate this hypothesis, we employed two strategies: one involved triggering oxidative stress through the exogenous addition of H₂O₂, while the other focused on regulating the expression levels of the endogenous catalase gene, *catA*, using CRISPR-Cas9 gene-editing technology.

Previous studies have shown that the exogenous addition of H₂O₂ at concentrations ranging from 1 to 100 mM significantly enhances CPC production in the wild-type strain *A. chrysogenum* ATCC 11,550 (Bibián et al. 2020). In contrast, our study revealed that the addition of varying concentrations of H₂O₂ led to a significant reduction in CPC yield during shake flask fermentation of the high-producing industrial strain FC^3^-5-23. This observation contradicts earlier findings (Bibián et al. 2020). We hypothesize that this discrepancy may arise from (1) strain-specific differences: FC^3^-5-23, a high-yielding industrial strain derived from mutations, produces approximately 18,710 µg/g biomass of CPC (calculated based on 1.31 g/L CPC production and 70 g biomass/L) (Xu et al. 2022), nearly 50 times more than ATCC 11,550, which yields approximately 200–300 µg/g biomass and may exhibit a different response to H₂O₂; (2) elevated intrinsic ROS level in high-yielding strains. Previous studies have reported that higher oxygen consumption leads to higher level of ROS in cells (Baez and Shiloach. 2014). Based on results from the 50 L fermentation process, which demonstrated high oxygen uptake rates (OUR), we postulate that the FC^3^-5-23 strain already maintains elevated intracellular ROS level. The exogenous addition of H₂O₂ may lead to a rapid increase in intracellular ROS concentration, surpassing the oxidative stress tolerance threshold of this strain, thereby inhibiting cell growth and reducing CPC biosynthesis efficiency. This aligns with the conclusions of Bibián et al. (Bibián et al. 2020), who reported that the impact of H₂O₂ addition is inversely correlated with the strain’s natural ROS level. Strains with lower baseline ROS level exhibit greater regulatory flexibility, whereas strains with elevated ROS level may experience rapid accumulation of ROS beyond cellular tolerance limits when subjected to exogenous H₂O₂, triggering oxidative stress and impairing both growth and production.

Furthermore, the addition of exogenous H₂O₂ significantly reduced catalase activity in the FC^3^-5-23 strain. This reduction may be attributed to oxidative stress caused by ROS accumulation, which can inhibit cell growth and impair cellular functions. Additionally, it has been demonstrated that the glutathione reductase-encoding gene *glrA* plays a pivotal role in managing H₂O₂-induced oxidative stress in *A. chrysogenum* CGMCC 3.3795 (Long et al. 2012). Therefore, the presence of exogenous H₂O₂ may have competitively inhibited the expression of the *catA* by inducing the overexpression of *glrA*, consequently reducing catalase activity.

Overexpression studies of the *catA* demonstrated that the PA1-cat exhibited enhanced tolerance, reduced apoptosis, and sustained higher metabolic activity under low-oxygen conditions, thereby stabilizing CPC production. These findings have significant implications for industrial applications: first, costs associated with oxygen supply can be minimized by controlling lower dissolved oxygen level during fermentation; second, overexpression of the *catA* enhances the strain’s ability to withstand unexpected challenges, such as mixing failures, aeration blockages, or power outages. Due to its increased tolerance to hypoxia, these unforeseen events do not adversely affect the fermentation process, decrease CPC production, or lead to interruptions. Finally, the combination of reduced apoptosis and sustained high metabolic activity contributes to enhanced fermentation stability.

In this study, the multifaceted roles of catalase in CPC fermentation were systematically investigated for the first time through the exogenous addition of H₂O₂ and the endogenous regulation of *catA* expression. Specifically, catalase regulates oxidative stress, reduces apoptosis, promotes mycelial differentiation, enhances hypoxia tolerance, and stabilizes CPC production. However, the precise regulatory mechanisms of catalase and its encoding gene, *catA*, in apoptosis, mycelial differentiation, and CPC biosynthesis remain unclear and require further investigation. Additionally, the PA1-cat strain did not reverse the significant negative impact of dissolved oxygen limitation on CPC biosynthesis. Analyzing the molecular mechanisms underlying dissolved oxygen limitation on CPC biosynthesis, alleviating feedback inhibition through engineering modifications, and enhancing the affinity for oxygen under hypoxic conditions are all viable strategies to improve CPC production in the future. Moreover, the reason for the elevated secretion of catalase in the industrial strain of *A. chrysogenum* remains unclear. We hypothesize that catalase may function synergistically with glucose oxidase to convert glucose into gluconic acid, thereby influencing CPC biosynthesis by altering carbon source utilization. Additionally, catalase, located in the peroxisome, might indicate peroxisome accumulation when secreted in larger amounts, which could enhance redox homeostasis in high-yielding strains. This phenomenon aligns with the rapid oxygen consumption required for CPC biosynthesis and the prompt removal of reactive oxygen species (ROS) byproducts. Furthermore, the involvement of peroxisomes in CPC biosynthesis in *A. chrysogenum* suggests that an increase in peroxisomes may accelerate CPC biosynthesis.

In the future, further in-depth studies on the multiple roles of catalase in stabilizing CPC production, as well as the mechanisms underlying its high expression in industrial strains, will enhance our understanding of the regulatory network governing catalase and CPC biosynthesis. These studies will also provide a theoretical foundation for strain improvement and fermentation process optimization. Overall, this study provides new avenues for enhancing industrial strains of *A. chrysogenum* and refining CPC production processes, with promising industrial applications.

## Conclusion

The efficient production of CPC by *A. chrysogenum* faces DO-limiting problems. The strategic addition of surfactants effectively mitigated mass transfer limitations, resulting in increased DO level and enhanced extracellular catalase content, which ultimately improved CPC production. Notably, we also observed a significant secretion of catalase during the fermentation of an industrial strain of *A. chrysogenum* for the first time. The addition of exogenous H₂O₂ resulted in reduced catalase activity and CPC production. The knockout of the catalase-encoding gene *catA* led to decreased DCW, intracellular catalase activity, H₂O₂ tolerance, and CPC production, as well as increased cellular morphological abnormalities and apoptosis. Overexpression of *catA* significantly increased the transcription level of catalase and intracellular enzyme activities, attenuated apoptosis caused by ROS accumulation, accelerated mycelial morphological differentiation, and enhanced oxidative stress tolerance under DO limitation in *A. chrysogenum*, thereby markedly stabilizing CPC yield during the late fermentation period. In this study, we systematically investigated, for the first time, the multiple functions of the *catA* gene in oxidative stress, apoptosis, mycelial differentiation, and hypoxia acclimatization. We focused specifically on the crucial role of *catA* in regulating metabolic activity and stabilizing CPC yield under oxygen-limited conditions. These findings provide significant theoretical support for the rational modification of industrial strains and the optimization of fermentation processes.

## Electronic supplementary material

Below is the link to the electronic supplementary material.

Supplementary Material 1

## Data Availability

All data generated or analyzed during this study are included in this article.

## Abbreviations

CPC: Cephalosporin C
DO: Dissolved oxygen
H₂O₂: Hydrogen peroxide
ROS: Reactive oxygen species
PMV: Packed mycelium volume
7-ACA: 7-aminocephalosporanic acid
OUR: Oxygen uptake rate
DCW: Dry cell weight
PI: Propidium iodide
PBS: Phosphate buffered solution
SDS-PAGE: Sodium dodecyl sulfate polyacrylamide gel electrophoresis
HPLC: High performance liquid chromatography
EO: Ethylene oxide
PO: Propylene oxide
PMSF: Phenylmethanesulfonyl fluoride
DCFH-DA: 2, 7-Dichlorodihydrofluorescein diacetate
CER: Carbon dioxide evolution rate
TPM: Transcripts per million reads
CFD: Computational fluid dynamics
