# Supplementary material for: Effect of catalase on CPC production during fermentation of Acremonium chrysogenum
Source: Bioresour Bioprocess. 2025 Jan 4;12(1):1. doi: 10.1186/s40643-024-00831-y (PMC11699191; doi:10.1186/s40643-024-00831-y)
Supplement: Supplementary file 1 — Supplementary Material 1 [file 40643_2024_831_MOESM1_ESM.docx]

Effect of catalase on CPC production during fermentation of *Acremonium chrysogenum*

Ling Liu^1†^, Zhen Chen^1†^, Xiwei Tian^1^* and Ju Chu^1^*

1 Qingdao Innovation Institute of East China University of Science and Technology, State Key Laboratory of Bioreactor Engineering, East China University of Science and Technology, 130 Meilong Road, Shanghai, 200237, People’s Republic of China

^†^ Ling Liu and Zhen Chen contributed equally to this work

* Corresponding author: Xiwei Tian and Ju Chu

Tel: +86-21-64253021/+86-21-64253853

E-mail address: xiweitian@ecust.edu.cn (XW. Tian), juchu@ecust.edu.cn (J. Chu)

# Supporting Tables

**Table S1** Peptide segment results of unknown proteins identified by mass spectrometry

**Table S2** Protein identification results of unknown proteins by mass spectrometry

# Supplemental Figure

**Fig. S1** The construction process of homologous recombination donor plasmids T-PA1-cat and T-PcefG-cat for *catA* gene promoter replacement. The arrows represent the primers, and the promoter can be either PA1 or PcefG. The constructed plasmids are utilized in subsequent PCR to obtain the homologous recombination donor DNA fragments, which are generated using primers cat-UH-F and cat-DH-R through PCR amplification. These donor DNA fragments function in conjunction with a knockout plasmid containing Cas9 and sgRNA during protoplast transformation, enabling homologous recombination to replace the native *catA* gene promoter with either the strong or weak variant

**Fig. S2** Effect of surfactant addition time, concentration and species on CPC production from FC^3^-5-23 shake flask fermentation. CK is the control check group. Different RD numbers correspond to different surfactants, with detailed descriptions provided in the Materials and Methods section. (a) Effect of adding 1% concentration of different species of surfactant at 72 h on CPC production; (b) Effect of adding 1% concentration of different species of surfactant at 72 h on PMV (c) Effect of adding 0.3% concentration of different species of surfactant at 60 h on CPC production; (d) Effect of adding 0.3% concentration of different kinds of surfactants at 60 h on PMV

**Fig. S3** The effect of H₂O₂ addition on extracellular catalase content, extracellular proteins were normalized using equal volumes of samples following centrifugation, with distinct bands observed above 70 kDa. (a) At 0 h of fermentation; (b) At 72 h of fermentation; Lane 1: control, no H₂O₂ added; Lane 2-3: 30 mM H₂O₂ added; Lane 4-5: 60 mM H₂O₂ added; Lane 6-7: 120 mM H₂O₂ added; Lane 8: 240 mM H₂O₂ added

**Fig. S4** Effect of exogenous H₂O₂ addition on extracellular catalase protein content during fermentation The catalase protein content was quantified using a semi-quantitative protein analysis method

**Fig. S5** Differences in catalase expression between high- and low-yield strains. *AcAxl2* is a regulatory factor involved in the hyphal septation process and arthrospore formation. Our previous research demonstrated that knocking out *Acaxl2* in FC3-5-23 led to a nearly threefold increase in CPC yield (Xu et al. 2022), referred to as the strain “Ac-Acaxl2::eGFP” in the figure. (a) Comparison of TPM value between 72 h and 144 h. TPM value, transcripts per million mapped reads or fragments value, is one of the commonly used algorithms for normalizing transcriptomic expression levels. A higher TPM value corresponds to a higher transcription level of the gene; (b) Comparison of *catA* transcription level between 72 h and 144 h., determined by qPCR analysis; (c) Comparison of SDS-PAGE results of extracellular proteins between 72 h and 144 h; (d) Comparison of intracellular catalase activity between 72 h and 168 h

**Supplemental tables**

**Table S1** Peptide segment results of unknown proteins identified by mass spectrometry

| Annotated sequence | Master protein accessions |
| --- | --- |
| [K].ITHFDHER.[V] | KFH49103.1 |
| [K].TKEEAEAILKPFADAVNK.[I] | KFH44965.1 |
| [R].EILLPGHCEEAGYDDWQGK.[Y] | KFH46238.1 |
| [K].GSAGLWDVHFR.[V] | KFH48940.1 |
| [K].TPETETKPNPECIGAFMLLHVTK.[S] | KFH48940.1 |
| [R].TVALTYTPSTPEEGIVR.[E] | KFH40658.1 |
| [R].GATGEDPPDSEYKEYLTAEDLK.[I] | KFH43146.1 |
| [K].GAWLYGTASEHNVLYNYHLQNAK.[N] | KFH48940.1 |
| [R].FETSHISPAIQENVLSQLNK.[I] | KFH49103.1 |
| [R].GATGEDPPDSEYKEYLTAEDLKIEEDGTSK.[M] | KFH43146.1 |
| [K].TGILTDPEGFDR.[A] | KFH42474.1 |
| [R].QVGAGFMEMMLLAAGPAGDR.[V] | KFH43146.1 |
| [K].LPTIATLHVGVLASSK.[S] | KFH49103.1 |
| [K].EFVPGEVTLSESDDPSQGASAASGK.[D] | KFH43136.1 |
| [K].AADIFAEELK.[V] | KFH42474.1 |
| [R].MLVFVHDPTDTEVIPVGK.[Q] | KFH40945.1 |
| [K].DLTPGNWVNR.[Q] | KFH46238.1 |
| [R].DGIVLLR.[N] | KFH43136.1 |
| [K].RGDHDNVLVLGGHSDSVAAGPGINDDGSGTIGVLR.[V] | KFH45625.1 |
| [R].NPSLQAAEEFIKR.[T] | KFH47162.1 |
| [K].RGATGEDPPDSEYKEYLTAEDLK.[I] | KFH43146.1 |
| [K].YSSEPLTINTQADPGEVK.[C] | KFH45377.1 |
| [K].KPAALLDDSGKFFVR.[S] | KFH48940.1 |
| [R].TGVGSEVWNVAK.[D] | KFH44965.1 |
| [K].EYLTAEDLKIEEDGTSK.[M] | KFH43146.1 |
| [K].ALAWYK.[D] | KFH48594.1 |
| [R].ILAAWYK.[M] | KFH43136.1 |
| [R].LHLIAETLDPITTEPAK.[M] | KFH47162.1 |
| [K].AESALGGLSLSDK.[V] | KFH43136.1 |
| [R].TPSFPAEGAPGIAMHPPSAMGKPASNR.[G] | KFH40642.1 |
| [R].ITLDVTEDVR.[L] | KFH40945.1 |
| [K].DFGFNMVR.[K] | KFH40945.1 |
| [R].SFGVVMFR.[G] | KFH47162.1 |
| [K].VLLHFEAVDYR.[S] | KFH40945.1 |
| [R].STGAGGLAIWTHGLK.[E] | KFH44965.1 |
| [R].DLSNWDVSK.[Q] | KFH48934.1 |
| [K].LIPTDTLTYTPSGQAK.[A] | KFH45625.1 |
| [K].LPLTAGESGTATFNLR.[K] | KFH43136.1 |
| [R].AAADLGIPEDADLQDLDSNHAVQK.[N] | KFH42474.1 |
| [K].EDAEPIIAALK.[E] | KFH45625.1 |
| [R].GYSDQVELFEPPFK.[T] | KFH43146.1 |
| [R].GIENNIWLLR.[Q] | KFH41508.1 |
| [R].FASSITAFTPSVQAASTWDR.[E] | KFH43136.1 |
| [K].GENNWFTNQNNFFR.[Q] | KFH48940.1 |
| [R].FETSHISPAIQENVLSQLNKISHDIATR.[V] | KFH49103.1 |
| [K].APLVLVDNLGCEESDFPAEVK.[D] | KFH45625.1 |
| [K].VIDIWPELTR.[T] | KFH45305.1 |
| [K].VADLSVVAHNVAANTASMALAIGEK.[A] | KFH42474.1 |
| [R].FAFWGAEEFGK.[L] | KFH45625.1 |
| [R].NYDFVWDFEK.[A] | KFH43146.1 |
| [R].FLSTGSFLLYPYAR.[G] | KFH42474.1 |
| [R].GPTLLEDFIFR.[Q] | KFH49103.1 |
| [R].FMLGDLEDADFASALEK.[G] | KFH46238.1 |
| [K].LDVNPINYFAEVEQAMFQPGHIVR.[G] | KFH49103.1 |
| [K].IIPEELVPLTPLGVLK.[L] | KFH49103.1 |
| [R].DLESILNVEEVGSALR.[A] | KFH43146.1 |
| [K].DRFMLGDLEDADFASALEK.[G] | KFH46238.1 |
| [R].LFYNSLTEVEQQFLIDAIR.[F] | KFH49103.1 |
| [K].MVVVSAGTLGTPLLLER.[S] | KFH42474.1 |
| [R].DDTRYESILNNYAWER.[T] | KFH48934.1 |
| [K].VPEEDPTYYHDNTTAGLSIFGNK.[L] | KFH49103.1 |
| [R].DLAVWNVEGK.[Q] | KFH43136.1 |
| [R].LFSYLDTQLNR.[H] | KFH49103.1 |
| [K].RGYSDQVELFEPPFK.[T] | KFH43146.1 |
| [R].NPSLQAAEEFIK.[R] | KFH47162.1 |
| [K].AKDFVSQLTLLEK.[V] | KFH48934.1 |
| [K].DFQETFYGANYR.[D] | KFH44965.1 |
| [K].VAGAEVAQLYLSYPESAADQPPK.[T] | KFH43136.1 |
| [R].RIDQFIYPYLNTASSGK.[E] | KFH48934.1 |
| [R].TGAVPFGNSGPDYPVFR.[N] | KFH48940.1 |
| [R].YESILNNYAWER.[T] | KFH48934.1 |
| [R].DKGVDVVLGPAIGALGR.[N] | KFH48934.1 |
| [R].NHEELIFYVGGAQ.[-] | KFH45377.1 |
| [R].TVEDQPDINFSSWTR.[D] | KFH48934.1 |
| [K].YVTSEALQQHITLDSLLAGSQK.[L] | KFH45625.1 |
| [R].GIDFTDDPLLQGR.[L] | KFH49103.1 |
| [R].GLGVHVLLAPVAGALGK.[I] | KFH43136.1 |
| [R].NQGDILPLK.[N] | KFH43136.1 |
| [K].EGLATFKFTNR.[F] | KFH49103.1 |
| [R].DVGIDLNR.[N] | KFH43146.1 |
| [K].ETGGAVVIMEHR.[Y] | KFH46238.1 |
| [R].DVHGFATR.[F] | KFH49103.1 |
| [R].GSADTARDVHGFATR.[F] | KFH49103.1 |
| [R].KRPSSFADHWSQPR.[L] | KFH49103.1 |
| [R].VAGIIEHEPR.[A] | KFH47162.1 |
| [K].KTPVFIR.[F] | KFH49103.1 |
| [K].SYTTLQR.[L] | KFH46238.1 |
| [R].GDVAIVGGK.[N] | KFH40499.1 |
| [R].SKPQYADVPVEK.[F] | KFH48940.1 |
| [R].AGSLSWSSGGVEAK.[V] | KFH43498.1 |
| [K].RPSSFADHWSQPR.[L] | KFH49103.1 |
| [K].LQDFADANDNHR.[A] | KFH45625.1 |
| [K].HFIANEQEHFR.[Q] | KFH48934.1 |
| [K].VVTAAHCSEGQDPASVSVR.[A] | KFH43498.1 |
| [R].HGGPNFEQLPINRPVVPIHNNNRDGAGQNFIHR.[N] | KFH49103.1 |
| [K].SDESLAEAR.[A] | KFH49103.1 |
| [R].LNALATTGR.[V] | KFH44216.1 |
| [R].HMDGFGVHTYR.[L] | KFH49103.1 |
| [K].KPAALLDDSGK.[F] | KFH48940.1 |
| [R].QKITHFDHER.[V] | KFH49103.1 |
| [K].ISHDIATR.[V] | KFH49103.1 |
| [R].ALKQAFAR.[D] | KFH49103.1 |
| [K].DRPTIVSR.[L] | KFH46238.1 |
| [R].IIHENEVDGR.[G] | KFH45377.1 |
| [K].YGAFFGSQQFTAR.[N] | KFH48940.1 |
| [K].ITHFDHERVPER.[A] | KFH49103.1 |
| [K].NKDGALPLKEPK.[F] | KFH48934.1 |
| [R].VGGSAGTQLQSDK.[C] | KFH48940.1 |
| [K].HYVGNEQELNR.[E] | KFH43136.1 |
| [R].QKITHFDHERVPER.[A] | KFH49103.1 |
| [R].LVTDDGKSKFVK.[W] | KFH49103.1 |
| [R].FSTVAGSR.[G] | KFH49103.1 |
| [K].GDGSTDDTEAINK.[A] | KFH48940.1 |
| [K].TVAQLNAR.[T] | KFH46238.1 |
| [R].VGSALGVK.[V] | KFH49103.1 |
| [R].DGAGQNFIHR.[N] | KFH49103.1 |
| [R].AYTGSEGEAVQATR.[D] | KFH48940.1 |
| [K].HYVGNEQELNRETMSSNIDEK.[T] | KFH43136.1 |
| [R].EQSPLLGR.[G] | KFH45482.1 |
| [K].GPVPFPGK.[H] | KFH45625.1 |
| [K].SIAYEQAENIK.[Q] | KFH46238.1 |
| [R].GLLIESTK.[G] | KFH48940.1 |
| [R].RAELGHPEPFPLR.[Y] | KFH43648.1 |
| [K].VRPTDEEIDAMGLSEGAR.[L] | KFH42474.1 |
| [R].GHVHITGPAIEDKIDLK.[T] | KFH42474.1 |
| [R].VNYNVIAETK.[R] | KFH45625.1 |
| [K].HLYSTDTNTNPLTYK.[S] | KFH46062.1 |
| [R].HGGPNFEQLPINRPVVPIHNNNR.[D] | KFH49103.1 |
| [R].LGLMVIQDMPSTRPDAQPNAEEHAEFRR.[Q] | KFH40945.1 |
| [K].NFKPPFDPSGR.[S] | KFH46238.1 |
| [R].GFFTAPNR.[E] | KFH49103.1 |
| [R].STDYAMGAWVK.[N] | KFH46062.1 |
| [K].NLDPWHGGNNLVK.[A] | KFH43136.1 |
| [K].ASLVWDEAQHMAGK.[N] | KFH49103.1 |
| [K].ASLVWDEAQHMAGK.[N] | KFH49103.1 |
| [R].IMAAYFK.[V] | KFH48934.1 |
| [R].GGPDNVIYFLADLLAAR.[A] | KFH43146.1 |
| [KR].VFIMSGIHAR.[E] | KFH43146.1 |
| [K].DGALPLKEPK.[F] | KFH48934.1 |
| [R].VAEALAHFK.[I] | KFH45625.1 |
| [R].TLPGAVVGDDPK.[V] | KFH43146.1 |
| [K].AVGVEIRPNPEHGNGAEVQTVK.[A] | KFH42474.1 |
| [R].RLDVFR.[G] | KFH42474.1 |
| [KR].VFLMSGIHAR.[E] | KFH43146.1 |
| [R].EASGPLLR.[K] | KFH49103.1 |
| [K].LNDIAYANGGNR.[A] | KFH40658.1 |
| [K].AAAHVLSVLAMDGKK.[L] | KFH40658.1 |
| [K].MKQDQGYPDTNLDRDVQGNHAQNVR.[A] | KFH43136.1 |
| [K].LIEGYQNGDETPK.[A] | KFH44612.1 |
| [R].NTESASGSDGRDVGIDLNR.[N] | KFH43146.1 |
| [R].YISPDGIR.[Q] | KFH42474.1 |
| [K].ESYPMDYYGKEGLGR.[T] | KFH46738.1 |
| [R].LVTDDGK.[S] | KFH49103.1 |
| [R].DTVMATYDTYR.[A] | KFH40599.1 |
| [R].LLLCVLGFMTFGYGSGEFEVTHDR.[L] | KFH47784.1 |
| [K].HSLSTEGRLYVDGSGARAPFGGK.[S] | KFH48295.1 |
| [K].ILKEEIGFR.[G] | KFH43136.1 |
| [K].EGLATFK.[F] | KFH49103.1 |
| [-].MSDSEGDERPYLICETQDTVR.[Y] | KFH40892.1 |
| [R].TNLGVGK.[M] | KFH46405.1 |

The amino acid sequences were annotated using LC-MS/MS. The raw data included molecular and fragment ion peaks, where molecular ion peaks correspond to peptide or protein mass, and fragment ion peaks result from cleavage during analysis, providing structural information. We then used Proteome Discoverer (version 2.1, Thermo Fisher Scientific, USA) to compare the data with the genomic protein sequences of *Acremonium chrysogenum* ATCC 11550 from the NCBI database, enabling accurate protein identification and annotation.

**Table S2** Protein identification results of unknown proteins by mass spectrometry

| # AAs | MW [kDa] | Gene ID | Molecular function | Coverage | Peptides | PSMs | Unique Peptides | Area: F1: Sample | Score sequest HT |
| --- | --- | --- | --- | --- | --- | --- | --- | --- | --- |
| 716 | 78.41 | ACRE_002550 | Antioxidant activity; catalytic Activity; metal ion binding | 47.6257 | 35 | 254 | 35 | 3.4E+10 | 598.46 |
| 452 | 48.77 | ACRE_061130 | Catalytic activity; metal ion binding | 33.18584 | 14 | 42 | 14 | 3.4E+09 | 110.66 |
| 720 | 76.25 | ACRE_060910 | Catalytic activity | 29.86111 | 15 | 30 | 15 | 3.3E+08 | 75.68 |
| 779 | 85.00 | ACRE_000180 |  | 22.59307 | 13 | 18 | 13 | 2.7E+08 | 46.34 |
| 609 | 66.19 | ACRE_067770 | Catalytic activity; nucleotide binding | 28.40722 | 11 | 14 | 11 | 83000000 | 33.19 |
| 507 | 53.65 | ACRE_035120 | Catalytic activity; metal ion binding | 30.57199 | 10 | 17 | 10 | 4.8E+08 | 38.51 |
| 879 | 95.92 | ACRE_000010 | Catalytic activity | 13.31058 | 11 | 15 | 11 | 70000000 | 28.43 |
| 278 | 30.07 | ACRE_019520 |  | 17.26619 | 5 | 11 | 5 | 4.4E+08 | 26.19 |
| 577 | 64.71 | ACRE_029520 | Catalytic activity | 18.19757 | 10 | 12 | 10 | 1.6E+08 | 20.44 |
| 622 | 70.37 | ACRE_083520 | Catalytic activity | 12.05788 | 5 | 7 | 5 | 79000000 | 9.80 |
| 592 | 63.36 | ACRE_042930 | Catalytic activity; nucleotide binding | 9.628378 | 4 | 5 | 4 | 38000000 | 4.19 |
| 460 | 49.99 | ACRE_038440 |  | 8.913043 | 3 | 3 | 3 | 30000000 | 6.74 |
| 493 | 52.46 | ACRE_086430 | Catalytic activity; metal ion binding | 8.924949 | 3 | 3 | 3 | 37000000 | 6.01 |
| 253 | 25.56 | ACRE_057750 | Catalytic activity | 13.04348 | 2 | 2 | 2 | 1.5E+08 | 5.32 |
| 648 | 71.25 | ACRE_030640 | Catalytic activity; metal ion binding | 4.012346 | 2 | 2 | 2 | 26000000 | 2.06 |
| 932 | 105.70 | ACRE_077770 | Catalytic activity | 1.072961 | 1 | 1 | 1 | 38000000 | 2.26 |
| 592 | 65.07 | ACRE_055490 | Catalytic activity | 2.195946 | 1 | 1 | 1 | 33000000 | 2.52 |
| 571 | 62.36 | ACRE_036780 | Transporter activity | 1.401051 | 1 | 3 | 1 | 2.7E+09 | 6.24 |
| 427 | 46.03 | ACRE_046070 | Catalytic activity; metal ion binding | 3.044496 | 1 | 1 | 1 | 18000000 | 0.00 |
| 681 | 76.04 | ACRE_038650 | Catalytic activity | 1.468429 | 1 | 1 | 1 | 11000000 | 0.00 |
| 474 | 51.49 | ACRE_024440 |  | 3.164557 | 1 | 1 | 1 | 27000000 | 0.00 |
| 403 | 42.17 | ACRE_049960 | Catalytic activity | 2.233251 | 1 | 1 | 1 | 12000000 | 2.20 |
| 376 | 41.82 | ACRE_086610 |  | 7.180851 | 1 | 1 | 1 | 3.8E+08 | 0.00 |
| 796 | 86.81 | ACRE_088170 | Catalytic activity; nucleotide binding | 1.130653 | 1 | 2 | 1 | 2.7E+08 | 2.82 |
| 290 | 32.85 | ACRE_003560 |  | 2.068966 | 1 | 1 | 1 | 50000000 | 1.73 |
| 1205 | 134.38 | ACRE_026820 | Protein binding | 0.580913 | 1 | 1 | 1 | 4.9E+08 | 1.74 |
| 77 | 8.96 | ACRE_084000 |  | 27.27273 | 1 | 1 | 1 | 1700000 | 0.00 |
| 888 | 98.34 | ACRE_007620 | DNA binding; metal ion binding | 2.59009 | 1 | 1 | 1 | 1.1E+08 | 0.00 |
| 1195 | 131.82 | ACRE_086970 | Protein binding | 0.920502 | 1 | 1 | 1 | 33000000 | 0.00 |
| 981 | 107.61 | ACRE_014550 |  | 2.446483 | 1 | 1 | 1 | 8.6E+08 | 0.00 |

Score sequest HT: a scoring system used in the SEQUEST HT (High Throughput) search engine for mass spectrometry data analysis. The Score Sequest HT is derived from several factors, including the number of matched peptides, the number of unique peptides, the number of peptide-spectrum matches (PSMs), and the sequence coverage. A higher coverage, combined with a greater number of peptides, PSMs, and unique peptides, results in a higher Score Sequest HT, reflecting greater reliability.

Peptides and Area: F1: Sample can reflect the protein content in the sample.

Coverage: The sequence coverage of the identified peptides.

Peptides: The number of different peptides identified, which indirectly indicates higher protein abundance.

PSMs (Peptide-Spectrum Matches): The number of spectra matched to the peptides.

Unique Peptides: The number of characteristic peptides specific to a protein.

Area: F1: Sample: The intensity of the protein in the sample. This can be used for quantitative comparisons between different samples.

**Supplemental Figures**


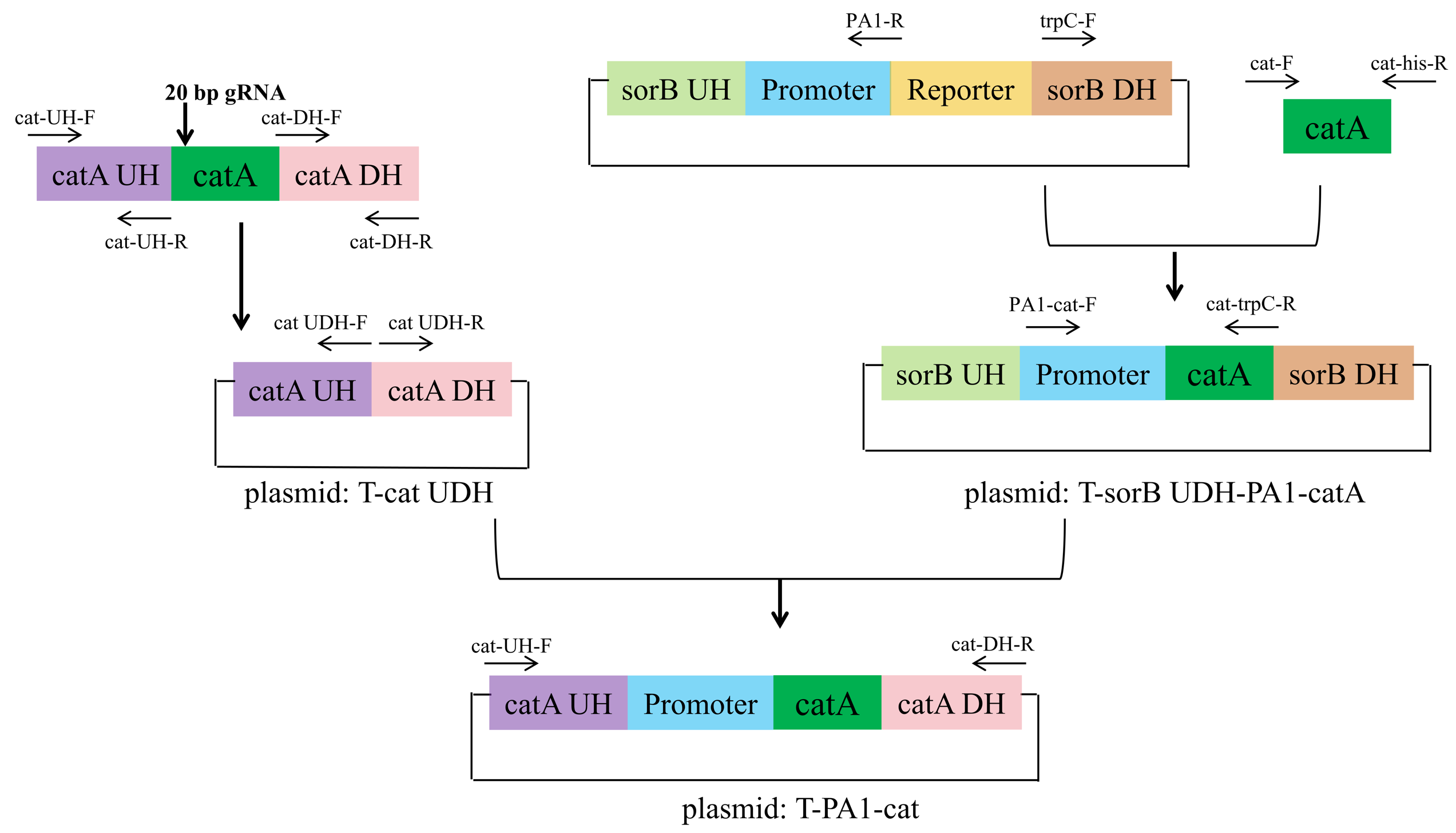


**Fig. S1** The construction process of homologous recombination donor plasmids T-PA1-cat and T-PcefG-cat for *catA* gene promoter replacement. The arrows represent the primers, and the promoter can be either PA1 or PcefG. The constructed plasmids are utilized in subsequent PCR to obtain the homologous recombination donor DNA fragments, which are generated using primers cat-UH-F and cat-DH-R through PCR amplification. These donor DNA fragments function in conjunction with a knockout plasmid containing Cas9 and sgRNA during protoplast transformation, enabling homologous recombination to replace the native catA gene promoter with either the strong or weak variant


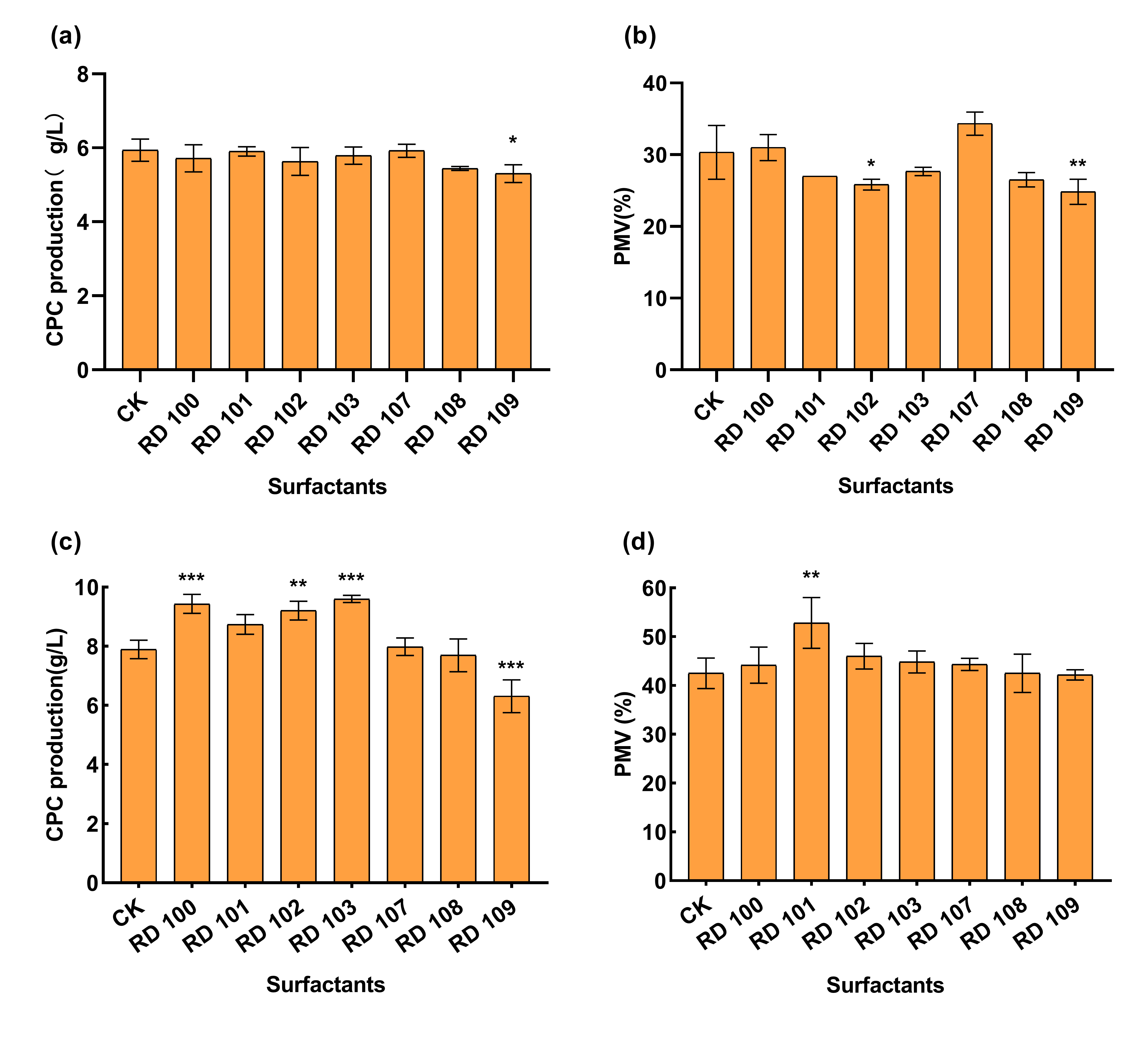


**Fig. S2** Effect of surfactant addition time, concentration and species on CPC production from FC^3^-5-23 shake flask fermentation. CK is the control check group. Different RD numbers correspond to different surfactants, with detailed descriptions provided in the Materials and Methods section. (a) Effect of adding 1% concentration of different species of surfactant at 72 h on CPC production; (b) Effect of adding 1% concentration of different species of surfactant at 72 h on PMV (c) Effect of adding 0.3% concentration of different species of surfactant at 60 h on CPC production; (d) Effect of adding 0.3% concentration of different kinds of surfactants at 60 h on PMV


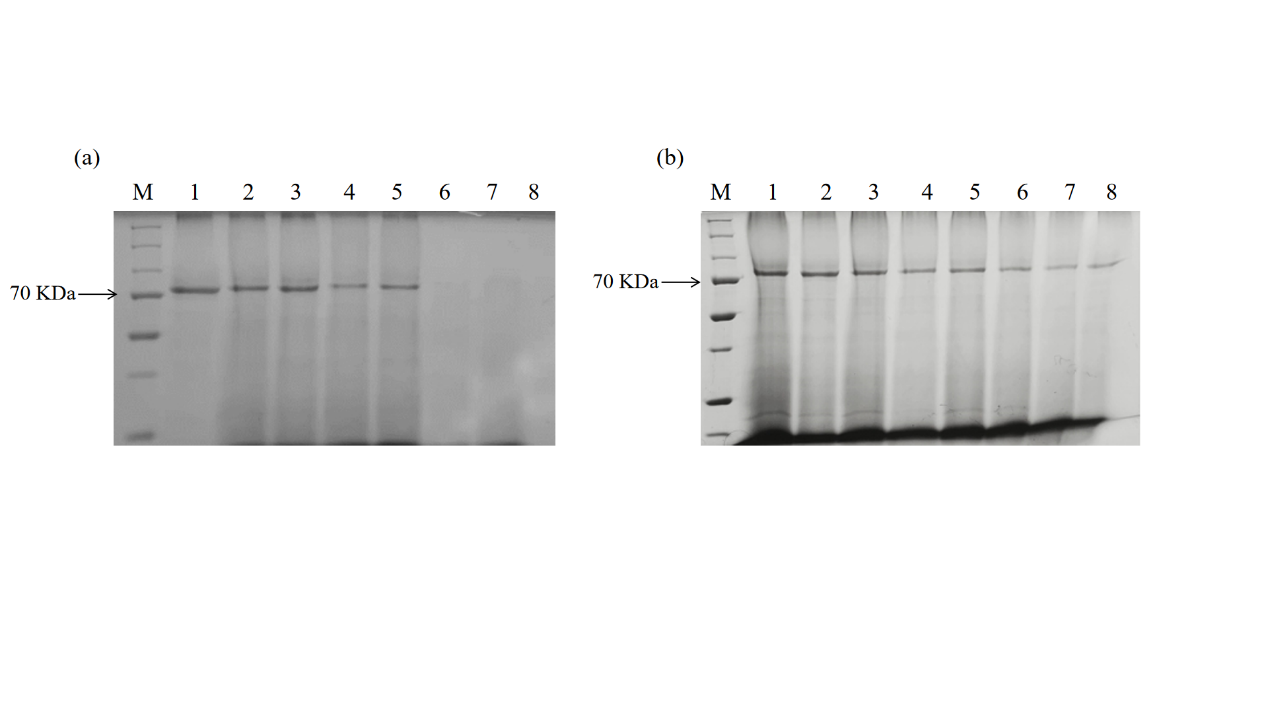


**Fig. S3** The effect of H₂O₂ addition on extracellular catalase content, extracellular proteins were normalized using equal volumes of samples following centrifugation, with distinct bands observed above 70 kDa. (a) At 0 h of fermentation; (b) At 72 h of fermentation; Lane 1: control, no H₂O₂ added; Lane 2-3: 30 mM H₂O₂ added; Lane 4-5: 60 mM H₂O₂ added; Lane 6-7: 120 mM H₂O₂ added; Lane 8: 240 mM H₂O₂ added


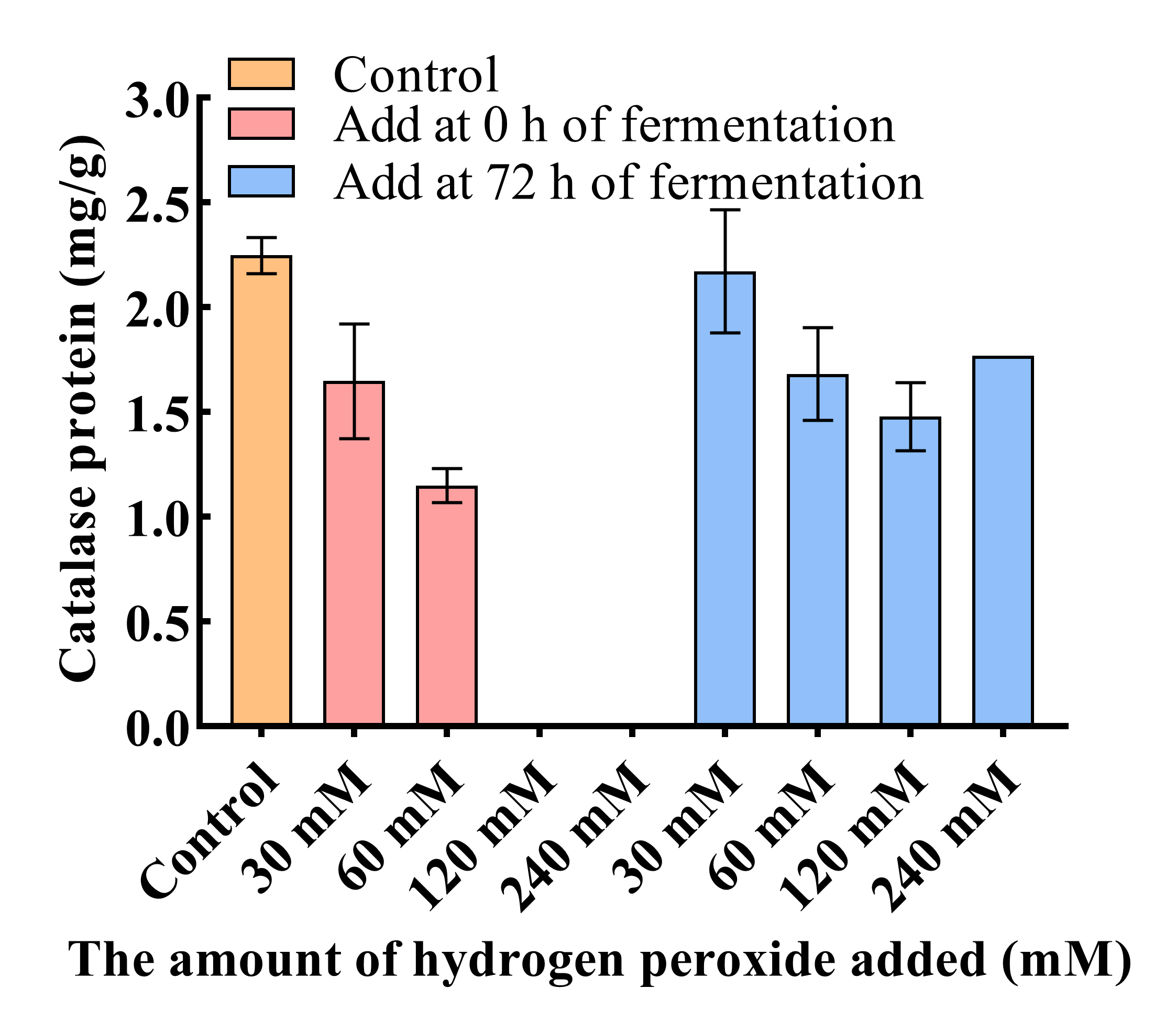


**Fig. S4** Effect of exogenous H₂O₂ addition on extracellular catalase protein content during fermentation. The catalase protein content was quantified using a semi-quantitative protein analysis method


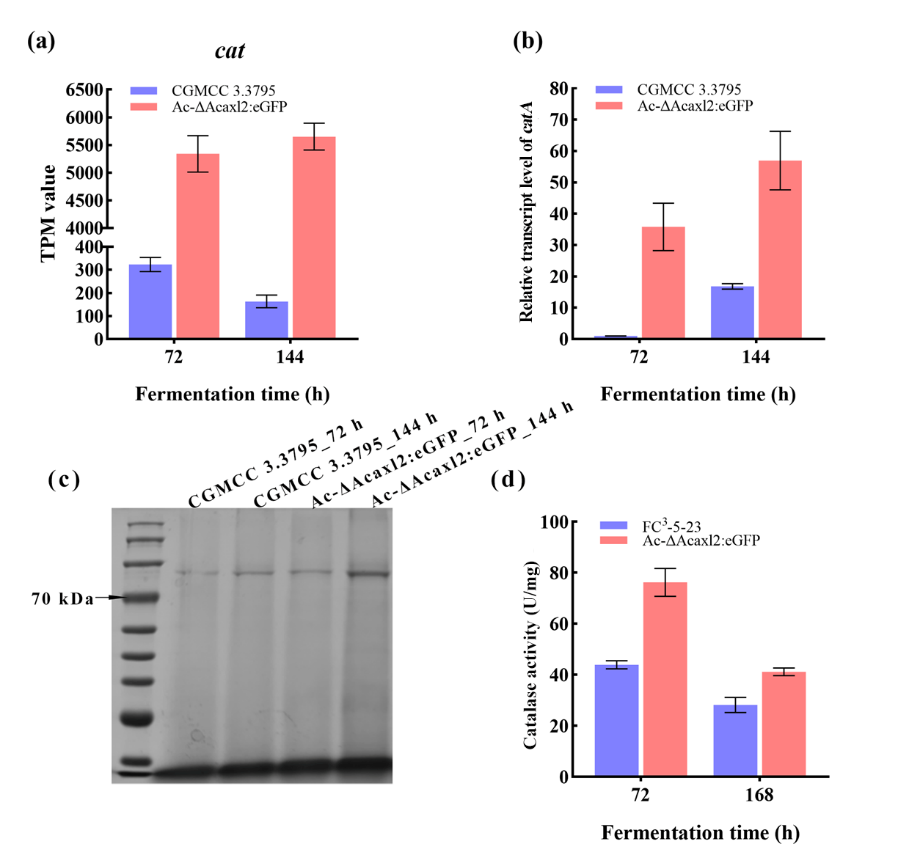


**Fig. S5** Differences in catalase expression between high- and low-yield strains. *AcAxl2* is a regulatory factor involved in the hyphal septation process and arthrospore formation. Our previous research demonstrated that knocking out *Acaxl2* in FC^3^-5-23 led to a nearly threefold increase in CPC yield (Xu et al. 2022), referred to as the strain “Ac-Acaxl2::eGFP” in the figure. (a) Comparison of TPM value between 72 h and 144 h. TPM value, transcripts per million mapped reads or fragments value, is one of the commonly used algorithms for normalizing transcriptomic expression levels. A higher TPM value corresponds to a higher transcription level of the gene; (b) Comparison of *catA* transcription level between 72 h and 144 h., determined by qPCR analysis; (c) Comparison of SDS-PAGE results of extracellular proteins between 72 h and 144 h; (d) Comparison of intracellular catalase activity between 72 h and 168 h
